# An evaluation framework and comparative analysis of the widely used learning management systems

**DOI:** 10.1371/journal.pone.0311111

**Published:** 2024-12-19

**Authors:** Adnan Abid, Abdullah Hassan Tufail, Osama Mohamed Ahmed Salem, Naeem A. Nawaz, Uzma Farooq, Irfan Abid, Kashif Ishaq

**Affiliations:** 1 Department of Data Science, University of the Punjab, Lahore, Pakistan; 2 School of Systems and Technology, University of Management and Technology, Lahore, Pakistan; 3 Dept. of Curriculum& Instruction, College of Education, Umm Al-Qura University, Makkah, KSA; 4 Department of Water Resources and Engineering, National University of Sciences and Technology, Islamabad, Pakistan; Najran University College of Computer Science and Information Systems, SAUDI ARABIA

## Abstract

Learning Management System (LMS) is a major tool used in most universities and institutions for online/distance education purposes. A variety of LM systems are being used in different universities and institutions, and these LM systems are also updating their versions and patches to stay in the competition. Hence, the selection of an appropriate LMS is an important task as it is going to influence the academic proceedings of an academic institution. There are a few online comparison sheets available for different LMS, which have been scored based on online feedback, but those online comparisons lack a comprehensive features list for the comparison, and are unable to provide a generalized scoring function to compute the suitability score for an LMS. This article proposes a framework to evaluate an LMS based on a detailed list of features and functions. This evaluation is scored through a generalized scoring function that computes the quantitative score of LM systems. Lastly, some widely used open access and proprietary LM systems have been evaluated using the proposed framework and scoring function.

## 1. Introduction

Modern education, when appropriately addressed, provides ample opportunities for collaboration, aligning with the dynamic requirements of technology. It seamlessly integrates traditional learning methods with technological advancements, offering educational support services that excel physical boundaries, catering to both on-campus and off-campus settings. On-campus resources encompass multimedia devices, smart boards, and smart desks, among others, enhancing the educational experience. In contrast, off-campus tools, such as online student portals and LM systems, play a pivotal role in meeting the demands of contemporary education.

According to a comprehensive study [1], an LMS not only delivers content but also efficiently manages course registration, administration, skills gap analysis, tracking, and reporting. Serving as a robust infrastructure, an LMS effectively delivers and oversees instructional content, identifying and evaluating individual and organizational learning or training objectives [2]. In the digital age, strategies for disseminating knowledge have gained prominence in higher education, transcending the confines of traditional face-to-face (F2F) instruction. The integration of e-learning with F2F teaching in university courses not only enhances accessibility and flexibility but also provides a myriad of choices for interactive learning experiences [3]. While LM Systems have undergone evolution, they remain a secure repository for content, ensuring exclusive access for enrolled students. These systems offer diverse avenues for facilitating in-built classroom communication, including the delivery of quizzes, assignment facilitation, and grade publication [4].

**Fig 1:**
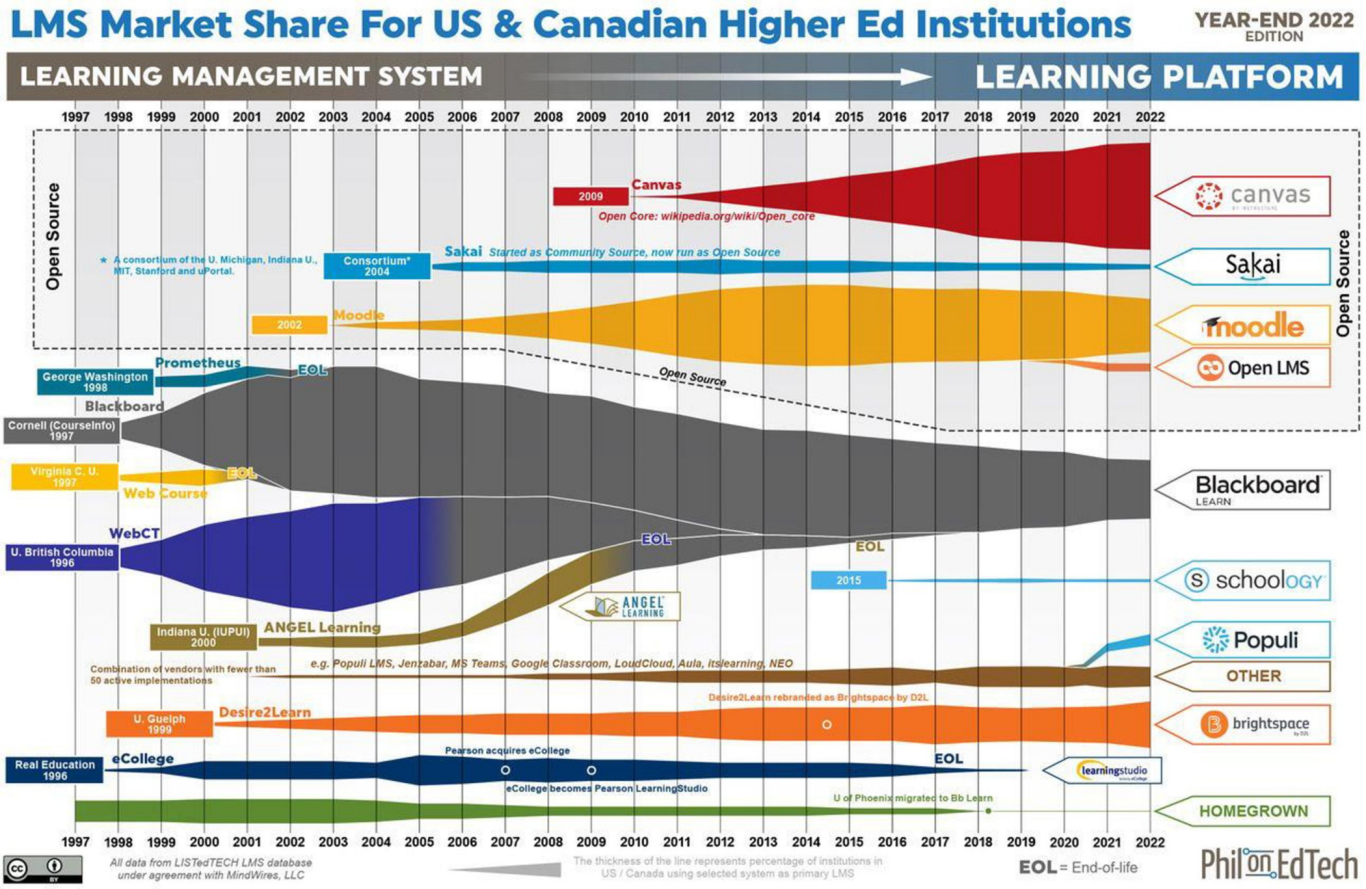
Higher education LMS market share for institutional adoption retrieved from: https://onedtech.philhillaa.com/p/state-of-higher-ed-lms-market-for-us-and-canada-year-end-2022-edition/.

Fig 1 illustrates the market share of Higher Education LMS concerning institutional adaptation. The data has been adjusted to encompass international usage and online programs, reflecting the growing influence of online education, notably massive open online courses (MOOCs), as a significant driving force in the future market. This summary is sourced from a combination of Campus Computing reports, ITC surveys, company press releases, and extrapolations based on Blackboard’s and Pearson’s quarterly earnings [5].

An LMS serves as a proactive and productive tool, pivotal in achieving the learning objectives set by stakeholders. Its primary aim is to facilitate students, fellow learners, and educators by offering a wide array of online educational resources in multiple formats—audio, video, documents, etc.—as well as enabling online social interaction. Hence, an instructor-friendly LMS should support instructors by enhancing their understanding and knowledge of instructional techniques.

**Fig 2:**
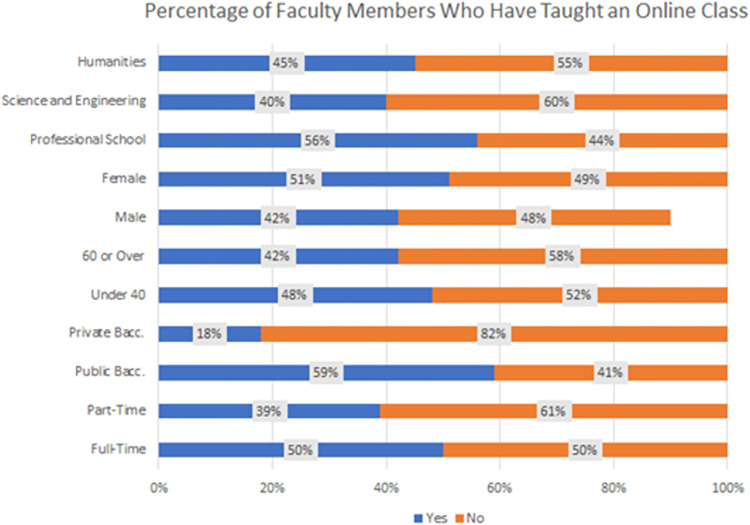
Percentage of instructors who have taught an online class.

Fig 2 portrays the percentage of instructors who have engaged in teaching blended or hybrid courses. The number of instructors employing a blend of in-person and online components has shown steady growth, rising to 44.54%. Approximately three-quarters of these instructors were actively involved in designing the blended courses.

LMS platforms are widely utilized across educational institutions globally, often integrating various learning management tools. For instance, Moodle, a prevalent LMS, features built-in modules for course management, file uploads, assignments, grading, and more. Additionally, tools like audio/video conferencing and plagiarism checkers, while third-party, seamlessly integrate with LMS platforms, fostering a collaborative solution. Some LMS are offered under the General Public License (GNU), allowing end-users to customize the source code based on their specific needs [6]. Some LM systems are available as a proprietary license software. This license offers one or more copies of licenses to end-users, but ownership remains with the software publisher. Different institutes adopt different LMS according to their requirements of functions/functionality and license agreements.

In the research article "What’s Next for the LMS?" [7], the discussion revolves around five domains of NGDLE (Next Generation Digital Learning Environment). Interoperability and Integration facilitate resource and tool exchange, while Personalization tailors the learning environment from individual to enterprise levels. Collaboration enables the movement of content across various spaces, and accessibility and universal design ensure global accessibility, providing ample opportunities for learning and growth [7].

**Fig 3:**
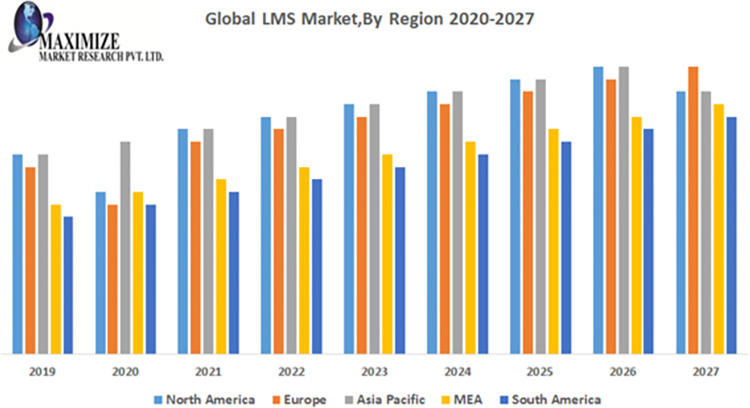
Global LMS market accounted for US$ XX Bn in 2018 and is expected to grow at a CAGR of 19.8% over the forecast period 2019–2027, to account for US$ 24.3 Bn in 2027 [9].

In a brief overview of Learning Management, Capterra’s LMS research (USA, UK, Canada) illustrates how modern, cloud-based LMS reduce training costs, enhance student satisfaction, and improve course completion rates as shown in Fig 3. As the world transitions to the cloud, 87% of respondents adopt web-based LMS, while only 13% rely on on-premise or installed systems [8].

An LMS serves as an online platform for delivering and managing e-learning courses [10], forming an essential part of the standard information technology infrastructure in higher education [11]. While e-learning systems predominantly facilitate instructor-student and student-student interactions [12], early Information Systems (IS) models often overlooked user interaction [13]. Researchers have emphasized the need for more comprehensive LMS strategies, emphasizing collaboration and intentionality to foster meaningful learning experiences [14]. To assess LMS quality from the students’ perspective in the arts, researchers used an information system success model to gauge student satisfaction [15]. This study aims to evaluate and compare the features and functions of various contemporary LMS on behalf of Binary Rubric, modularity of availability and analytic rubrics. Through the Feature-Rubric Scoring Function (FRSF) designed for selecting the best-fit LMS for university-oriented courses, the study developed a range of features, categorized and described in a defined rubric format. The study’s scoring function provides a unique method for assessing different LM systems, enabling the evaluation of new LMS based on their features and functions. The paper focuses on four specific LMS systems but allows for the inclusion of others in the evaluation category. The study [16] evaluates usability of LMS in the literature by utilization of multiple criteria decision making (MCDM) methods those are very rare in real life. Based on this motivation, employing axiomatic design procedure (ADP) is use at an academic department. This paper [17] has reviewed systematically between the current LMS, the problem with existing LMS to find out potential solutions that might help. Four learning management systems are chosen and findings from this review can be used to help users such as high schools, universities/colleges, and students select their LMS.

Evaluation of LMS is done in different perspective including, in clinical care, complex techno-social systems, identification of the elements necessary for inclusion in policy, Pedagogic and Strategic Resources, Technological Resources, most effective pedagogical tools and technologies, Plagiarism checking, and satisfaction level for various features and operational functions. An LMS evaluation framework by Berkley has been used to evaluate the ability of the package to meet the university’s current academic and administrative requirements and selection. Also, future requirements and selection are currently known to exist [18]. McGill has used an LMS evaluation tool and evaluated the set of criteria by scoring each item: 0 (Feature Not Present), 5 (Fair), 8 (Good), or 10 (Excellent) based on the information and demonstration provided by the vendor [19].

This paper discusses related work in the next section, and the evaluation framework is discussed after the related work. The proceeding section presents features and functions for the proposed framework, scoring function, and evaluation of the framework on LMS. Lastly, a discussion and overview of LM systems’ scoring rubric, conclusion, and future directions are presented.

## 2. Related work

Digital learning appears challenging in the arts discipline because artistic development is predominantly studio-based, and technology integration tends to be addressed within digital production courses [20]. There is a piece of evidence that differentiates degrees of usage influence, how LMS users perceive satisfaction, and the kinds of quality factors they value [21, 22]. A more refined understanding of the relationships between quality and satisfaction of LMS by usage differences is needed, as studies of students’ LMS preferences indicate their desire for more customization [23]. Understanding the users’ differences among art students can also help faculty optimize LMS features for personalization. A standard LMS support has been discussed for an inclusive learning environment for academic progress with interceding structures to promote online collaborative groupings, professional training, and communication among LMS users [24]. They investigated Learning Management System (LMS) factors that affect its self-efficacy and the impact it has on student satisfaction [25]. A study has been done to add knowledge to the extant literature for ensuring sustainable deployment of LMS in universities in Sub-Saharan Africa [26].

Similarly, a study has been done to enhance the online platform’s quality, succeed in LMS implementation, and facilitate the learners that need to be improved [27]. The proposed technique machine learning-based framework to capture students’ actions that indicate general leadership qualities and technical competence, as evidenced by their academic performances [28]. An Analysis of a Moment Structures (AMOS) as an analytical tool, Technology Acceptance Model (TAM) as research model, and the Structural Equation Model (SEM) to analyze the acceptance of LMS technology [29].A web-based LMS was built by using the PHP programming language and MySQL through the waterfall model method [30], whereas an LMS-based E-Learning system has been developed that is tested on microteaching in the mechanical engineering education class [31]. The work aims to consolidate these findings by evaluating surprisal estimates from Transformer-based language model variants that vary systematically in the amount of training data and model capacity on their ability to predict human reading times [32]. Researchers in different educational environments worldwide have used different models based on distinct criteria to investigate the acceptance of e-learning systems and technologies [33]. Evaluating the acceptance of LMS among the students’ beliefs regarding the system’s effectiveness for studying [34, 35]. The study revealed the factors influencing students’ satisfaction with an LMS to create a more effective learning environment [36]. By using the modified unified theory of acceptance and use of technology (UTAUT) model, they studied the factors which affect mobile banking (M-banking) acceptance in Islamic banks of Pakistan [37]. Likewise, research has been conducted to check the students learning activities by using LMS-based Moodle applications [38], whereas self-regulated learning in the workplace was introduced, which is an evidence-oriented curriculum reform. It is associated with higher student satisfaction and appears to support exploring which elements of an LMS most effectively help to achieve educational outcomes [39]. During the core clerkship year, difficulties can arise from becoming involved in the clinical work and actively participating; a negotiating technique has been adopted [40]. This indicates that to keep the learning process continued, using this technology during the COVID-19 pandemic is the need of the hour to continue the learning process. They argued that instead of complex variables (e.g., event logs, clickstream data, timestamps of learning activities), data extracted from online formative assessments should be the starting point for building predictive Learning Analytics (LA) models [41]. The primary objective of this study was to examine the factors affecting students’ learning motivation and academic performance during online learning using a novel framework of ergonomic appraisal [42]. the study aims to evaluate the success of learning management systems using the modified DeLone and McLean model. Statistical analysis was used to evaluate vocational teachers research model, specifically linear regression analysis [43]. This comprehensive review [44] identifies successful academic tools for online education that top universities have used in education. This study examines how to enable effective learning among all students by evaluating learning skills, e-flipped classrooms, e-problem-based learning, outcome-based teaching, assessment evaluation techniques and learning pedagogy.

A few examples of LMS used in educational institutions include Moodle, WebCT, Blackboard, and Desire2Learn [45–47]. It has become a common practice to use an LMS, especially in undergraduate medical education [48], primarily only convincing practical advantages rather than empirical evidence. In clinical care, working with information technology has become a core competency for graduating medical students [49–51]. The COVID-19 pandemic has increased his demand for flexible and creative use of virtual teaching [52]. A high mortality rate disease was high-infectious that has caused an increase of fear among people [53], as the worry regarding COVID-19 that might be infected the individual based on contact with patients [54]. As a result of the COVID-19 crisis, public policies that include social distancing, isolation, and self-quarantine have been implemented by governments worldwide to avoid unprecedented economic and psychosocial consequences worldwide [55]. The re-engineering and LMS development approach can be generally applied to speed many care process modifications, and improvement efforts have been studied. They included the patient flow and each team member’s role at every phase of the LMS [56].

The standard defined learning objects developed by the IEEE working group in the IEEE 1484.12.1, i.e., a 2002 Standard for Learning Object Metadata (LOM) which is defined as “*any entity*, *digital or non-digital that may be used for learning*, *education or training*” [57]. Summarizing the LOM standard aims to fulfill the purpose of well-structured and well-defined descriptions of learning resources and reduce the cost of high-quality providing resources. Moreover, modification of resource descriptions to fit the needs of the learning community culture, along with adding an extension to LMS so that developers could link learning resources with associated resource descriptions.

**Fig 4:**
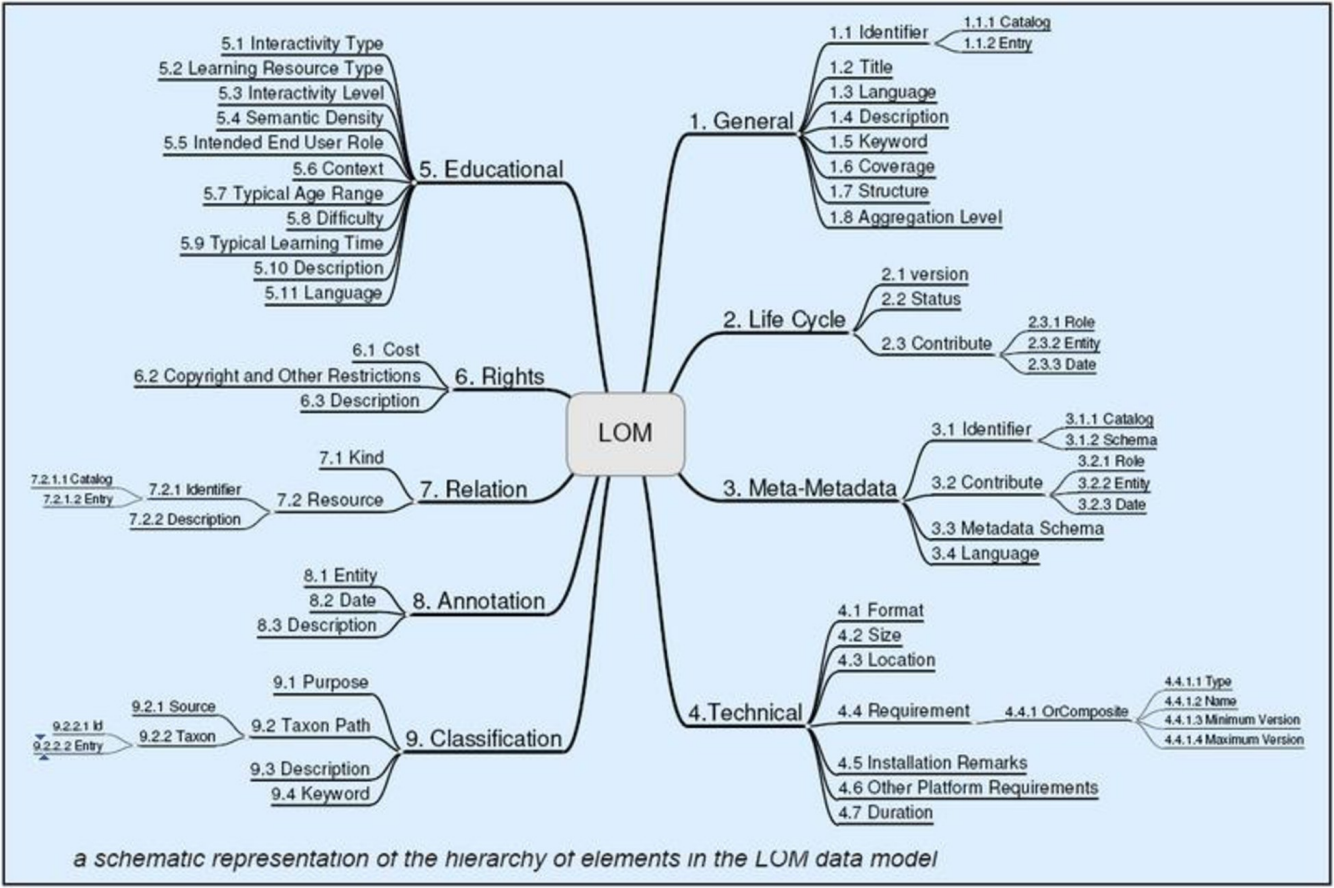
A schematic representation of the hierarchy of elements in the LOM data model–retrieved on 27-01-2021 from https://meta.wikimedia.org/wiki/learning_object_metadata.

They proposed that learning management system environments are complex techno-social systems that require dedicated standalone policies to regulate their operation. This preliminary study examined a selection of learning management system policies from twenty universities in four countries to identify some of the elements that are considered necessary for inclusion in policy documents [58]. Attending classes and sleeping well are important for students’ academic success. Here, The tested whether early morning classes are associated with lower attendance, shorter sleep and poorer academic achievement by analysing university students’ digital traces [59]. A questionnaire survey conducted by the South Korean research team of 163 e-learning experts regarding 81 validation items developed through a literature review was used to ascertain the importance of the criteria to validate a model for evaluating LMS used in e-learning fields [13] [60]. In this study, seven fields are defined to evaluate the LM system in which organizational demand is very important, without it, the other six categories could not be validated properly. The next four categories are core to e-learning and directly related to instruction. Further, the remaining aspects are not less valuable to e-learning. The summary of the seven fields is presented in Fig 4:

*Organizational demand*: Factors affecting LMS validation are the appropriateness of learning demand, economic validity, and user support.

*Instructional management*: It includes control of e-learning administration. This control comprises all aspects from user accessibility to LMS completion.

*Interaction*: It comprises communications in LMS. E-mails, chat rooms, and messages are included in this tool.

*Evaluation*: This module incorporates test management, easy use, and reusability of test modules in LMS.

*Information guidance*: Three subcategories are involved in validation. The first step is the easiness of information initiation, the second step includes information search-ability, and lastly, its accessibility.

*Screen design*: This aspect of LMS validation has to offer clarity of site direction, consistency, readability, and ease of navigation.

*Technology*: Its platform should be interoperable with other LMS platforms, and its data resources must have reusability and system stability [60].

A study [61] concluded the following core features in a Computer Science (CS) course in LMS: Software Engineering tools (video techniques, dedicated tools, animations); Classification of LMS content (production/development, live/presentation); user classes (consumers); authors (add/edit learning content) and administrative (management/ maintenance of learning portal). University courses have programming labs that can be standardized through the LM system. The study examines the educational process using learning management systems; the possibilities of descriptive, predictive and prescriptive types of analysis with the following selection of the most effective pedagogical tools and technologies for the successful acquisition of the required competencies by students [62]. A study in [15, 63] explained some essential core features for such a learning environment are given: Technological Resources (Virtual Communication, Simulator, Remote Lab, Virtual Machine, Automatic Assessment Tool); Pedagogic and Strategic Resources (Learning Methodology, Supporting and other Documentation, Evaluation); Academic Staff Resources (Teacher). Another study on computing education proposed the following fields in [64] for Moodle: Online Scripts & Exercises; Integration of Algorithmic Visualization; Automatic LE Assessment & Feedback; Interactive Compiling & Debugging Systems; Program Plagiarism Detection Support; and Personalization of Documents in LMS, e.g., edit/highlight texts.

It is proposed in [65] that Personalized learning materials and scenarios must be available in a Java Programming Course. In addition, an adaptive e-learning system is proposed for students to adopt individually following their targeted knowledge level, background knowledge, learning styles, and interests for various topics [65]. They identified students who were likely to do poorly in a biology course and those who were likely to do well. Then, we randomly assigned a portion of the students predicted to perform poorly to a science of learning to learn intervention where they were taught self-regulated learning (SRL) study strategies [66]. The study [67] data taken from learning activity and scores were obtained from the LMS student tracking report and at the end-of-semester academic performance. The study showed that each online learning activity significantly predicted academic performance, in which correlation and hierarchical linear regression are analyzed. Programming code plagiarism checking is a tricky task for an evaluator, so a study [68] presents the following tasks made to check plagiarism in a programming assessment: Automated assessments of assignments were made, but in our study, we preferred semi‐automated assessment; The proposed system uses the well‐known Moss tool to detect source code plagiarism; The online compiler provides a considerable reduction in the time required for the grading process of programming assignments, and the Moss system prevents our students from cheating and increases their success in the Data Structures course [68]. A time series design was used for two different Advancity Learning Management Systems (ALMS) and Moodle LMS sessions. Moodle and ALMS both receive relatively similar assessment ratings for online exams, but Moodle is better in terms of learning setting [69]. The study [70] explores the transformative potential of AI technology in the field of education including applications, benefits, and challenges of integrating AI into LMS.

Plagiarism checking is an essential part of authenticating an assessment in a learning environment, but it is tricky for a programming assessment, as discussed in [71]. The findings of the study are as follows: Syntax checker to report errors from the compiler; Plagiarism detector to detect similarities among accepted source codes; Black Box tester communicates the target program via UNIX to compare the expected results for validity, and Fingerprint generator to records finger-print in order of “N” in a hash table.

**Fig 5:**
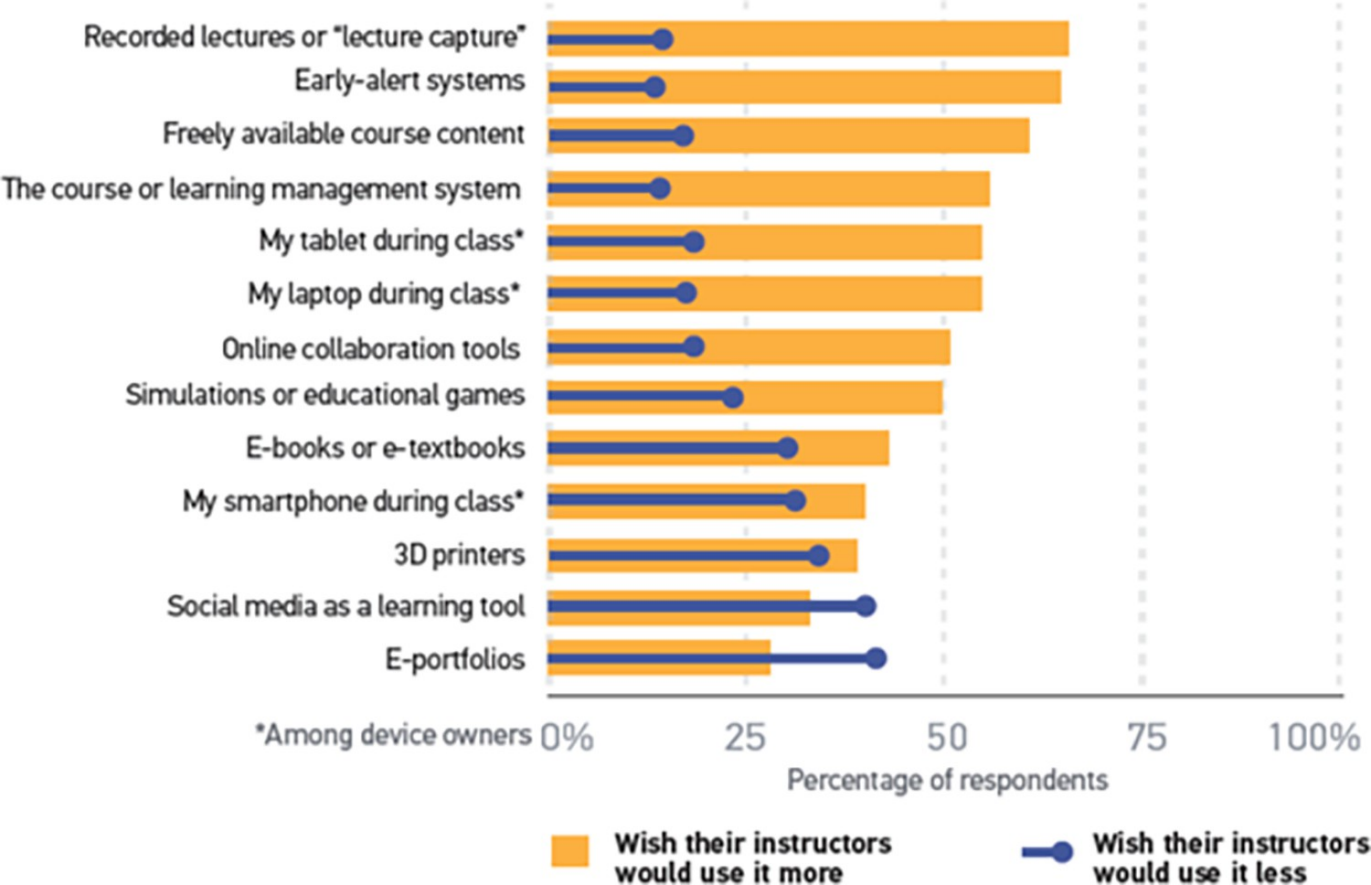
Percentage of students saying they wish their instructors would use technology more or less [72].

The study [72] stated students’ expectations for technology adoption by the instructors for the course were lecture capture, early-alert systems, and the freely available course content on the top of the list. Each technology has a low “use it less” companion percentage. Two technologies, social media as a learning tool and e-portfolios, had “use it less” rates that exceeded the “use it more” rates. Comparing these data from previous years, we see small but noticeable declines in nearly all “use it more” rates. E-portfolios and simulations/educational games were the only two technologies whose “use it more” rates increased (by three and two percentage points, respectively). Tech inclination is positively related to all items in the “use it more/use it less” question (r = .153–.297). In other words, the more tech-inclined students are, the more they wish their instructors would use these resources are presented in Fig 5 [72].

**Fig 6:**
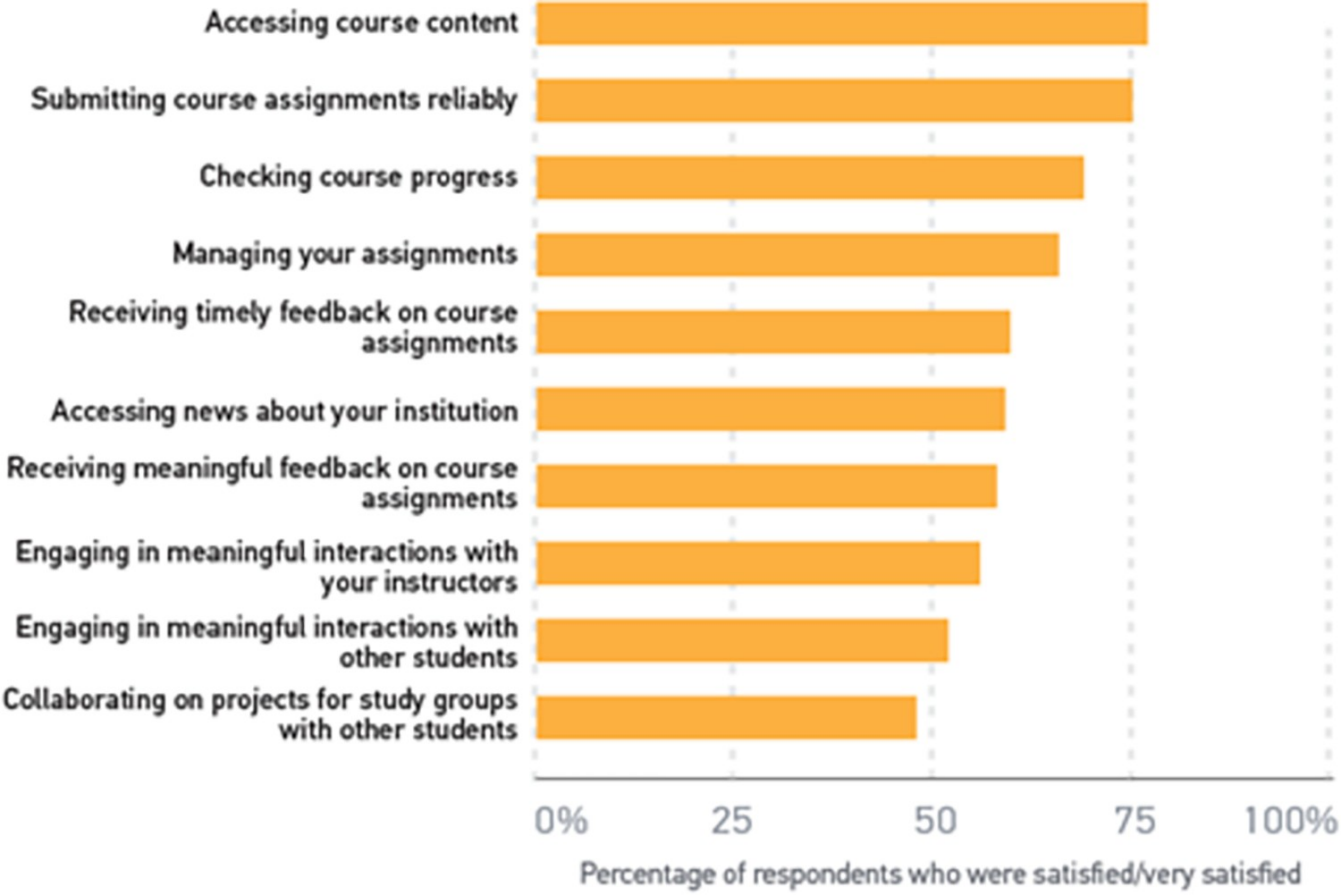
Overview of student satisfaction with LMS features and operational functions: [72].

**Fig 7:**
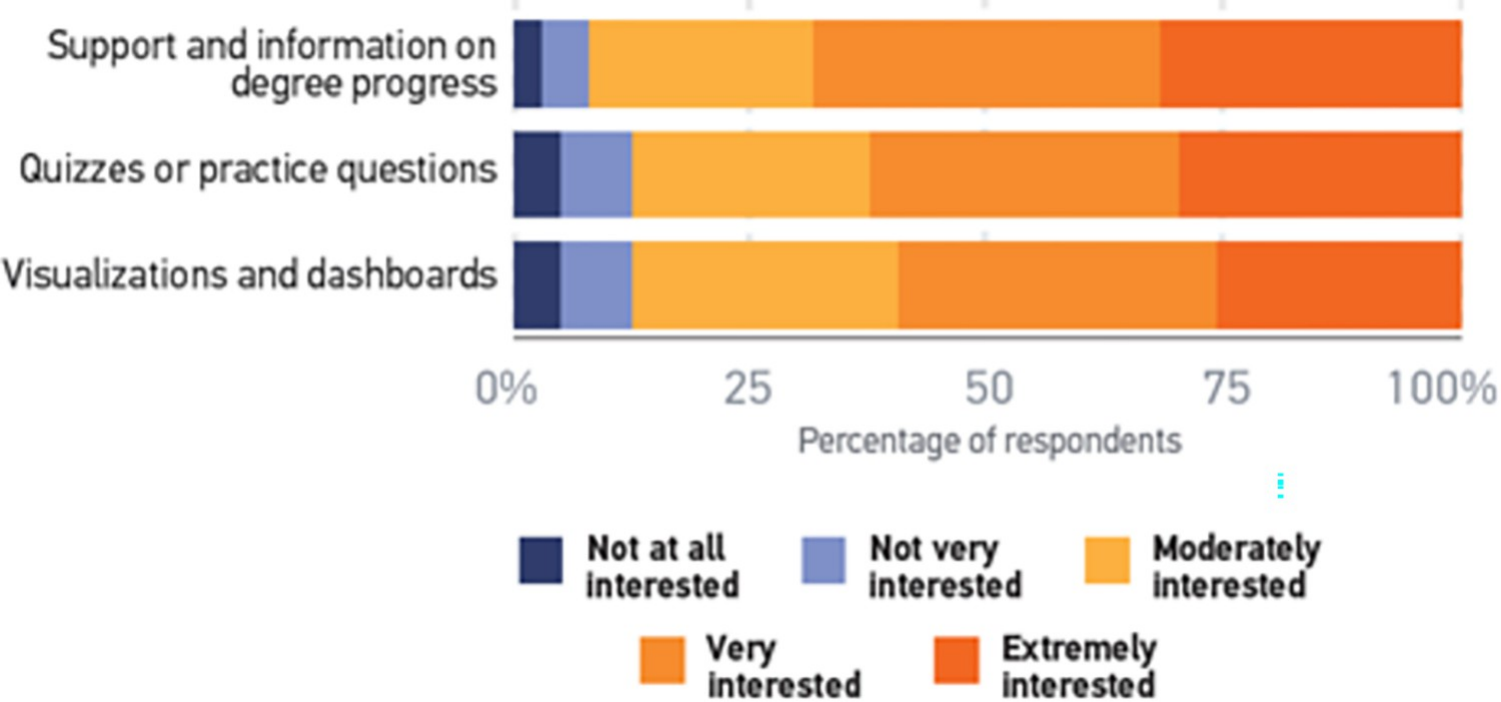
Students’ interest in personalized LMS features [72].

In the same research, ECAR also asked students about their satisfaction with various features and operational functions of the LMS. Satisfaction levels were highest for basic features such as accessing course content and lowest for advanced features such as using the LMS in engaging or collaborative ways. ECAR found similar results in the 2014 faculty study, with only about half of the faculty (51%) saying they were satisfied with the LMS to engage in meaningful interactions with students. Fig 6 presents a brief overview of student satisfaction with LMS:

Lastly, Fig 7 shows the interesting statistic of personalized LMS discussed in [24]. An additional area of interest concerns adaptive learning functions of the LMS, whereby students are provided with personalized quizzes or practice questions oriented to their specific strengths or weaknesses so that they (or their instructors) know what help they need (62%).

Exploring the analytics, students are quite interested in suggestions for improving performance, guidance about courses in the future alerts, feedback about their performance compared to that of other students, and automated tracking of their course attendance [72]. An LMS evaluation framework by Berkley has been used to evaluate the ability of the package to meet the university’s current academic and administrative requirements and selection. Also, future requirements and selection are currently known to exist [18]. McGill has used an LMS evaluation tool and evaluated the set of criteria by scoring each item: 0 (Feature Not Present), 5 (Fair), 8 (Good), or 10 (Excellent) based on the information and demonstration provided by the vendor [19].

The Berkeley [18] evaluation framework discusses more than 29 features and compares the Blackboard and WebCT LMSs on the basis of these features. McGill [19] designed an evaluation tool based on 27 features for generalized LMSs. This study, “Evaluation of Learning Management System (LMS) Though Feature-Rubric Scoring Function (FRSF) For Selecting Best Fit for University Oriented Courses,” has developed various features and functions in light of numerous literature reviews and by adhering to LOM (Learning Object Metadata) standards. The proposed evaluation framework in this study addresses seven well-structured main categories of features, 24 sub-features, and over 400 items/functions under these sub-features. Furthermore, it also defines the rubric to evaluate the conformity of each of these lowest granularity items (400+), which in turn helps in computing overall conformity score using a prescribed scoring function for an LMS in different levels of granularities.

This study also compares the features and functions of top-rated LMSs (including Moodle, Blackboard, TalentLM, and Canvas) using the defined rubrics and scoring function. It is pertinent to mention that the evaluation has been conducted using bounded and unbounded scores through the proposed scoring function. Thus, the proposed evaluation framework not only defines more than 400 evaluation parameters, but also provides a scoring function to compute conformity score for an LMS. Lastly, the proposed evaluation framework is generic and can be used to evaluate and compare different learning management systems.

## 3. Features, functions, and scoring for the proposed framework

This section covers the proposed framework for evaluable LMS’s features, and functions are generally discussed along with its scoring in the sub-section.

### 3.1. Features and functions for the proposed framework

The features and functions of top-rated LMSs are compared for rubrics to evaluate them accordingly, divided into seven departments and further sub-features set. These sub-feature sets are comprised of a collection of focused LMS functions. As it is discussed in previous sections, LOM, LMS core features, and evaluation models, this framework covers all prerequisites and can be compared. Table 1 shows the Features departments discussed in this paper for their sub-divided feature set and a number of functions.

**Table 1:** Features and functions of departments, sub-features, and their number of functions.

Features’ Department	Sub-Features Department	Number of Functions
**Platform and Architecture**	Interface, Platform & Structure	31
	Vendor’s Architecture & Integration	31
	Vendor’s Credentials	4
**Administration and Reporting**	Administration/Site Management	42
	Security	15
	Reporting Features	28
**Academic Support Features**	Talent Development & Certificate Tracking	3
	Workflow & Approval Process	5
	Instructor-led courses	7
	Evaluation	22
**Content Development and Management**	Learners’ Features	27
	Learning Content Management	62
	Learning Content E-Library	4
	Catalog & Search Feature	6
	Content Plagiarism	3
**Smart Features**	Mobile Features	13
	Gamification	17
	Virtual Classroom	15
**Learners’ Support Features**	Administration of Learner Management	16
	Social Media Features	25
	Communication & Collaboration Features	20
**LMS Support Tools**	LMS Plugins	7
	E-commerce Features	5
	Technical Support	4
*Total*: **7**	*Total*: **24**	*Total*: **416**

The features exhibit different behaviors, and to evaluate the conformance of a given LMS to a given feature, and we need to consider the underlying characteristics of a given feature. This leads us to define different feature categories regarding the conformance evaluation of considered LM Systems. After a thorough review, it is observed that these features can be categorized into three main types shown in Table 2. These main types are discussed briefly: 1) Binary Rubric in which features and functions are either available or not are set with a binary rubric, 2) Analytic Rubric in which few features have a separate scoring criterion that differs in range concerning numbers, and 3) The modularity of Availability that has four scoring functionality options. Two options are for feature availability, and the other two options are available via program script and availability via a plugin.

**Table 2:** Rubric types with descriptions.

#	Rubric Type	Description
1	Binary Rubric	The feature is not available
		Feature is available
2	Analytic Rubric	Rating of a score on each criterion of availability. Each criterion differs concerning features. Rating for each feature is analytically described in the rubric description.
3	Modularity of Availability	The feature is not available
		Feature available via program script
		Available via plugin
		Feature is available

### 3.2. Scoring function for the proposed framework

A framework of features and functions is briefed in the above section, which is evaluable via rubrics. These rubrics are combined to make a scoring function. In this section, we will evaluate LMSs by defining a simple scoring function that will support computing a quantitative score for each LMS. This score will be a reflection of the features and functionality strength of the respective LMS, given in Table 3.

**Table 3:** Mapping of qualitative measures into quantitative scores for the rubric.

Qualitative Measurements	Quantitative Measurements
**Binary Rubrics**	
Feature Available	1
Feature Not Available	0
**Modularity of Availability**	
Feature Availability (*Fully*)	1
Feature Availability via Plugin (*Mostly*)	0.66
Availability via Program Script (*Partially*)	0.33
Feature not Available (*No*)	0
**Analytic Rubric**	
Feature Availability range > x (*Fully*)	1
Feature Availability range b > x > c (*Mostly*)	0.66
Feature Availability range a > x > b (*Partially*)	0.33
Feature Availability range < x (*No*)	0

This paper has three types of rubrics for scoring purposes. First, Binary Rubrics fully maps compliance or availability to 1 and no compliance or availability to 0. Fully compliance means that the feature is seamlessly available in LMS, whereas No availability means that the feature is not offered in LMS. Second, Analytic Rubrics map the range of availability with respect to scored numbers. This range of availability differs concerning features in this paper i.e., each feature differs in its analytic rubric description. For example, if only a single language is available for multiple languages feature in LMS, then the rubric description sets the score as 0. If it ranges 1<x<6, then it is available Partially, and if it is available in a range 5<x<11, then it’s available Mostly. Similarly, if it ranges x>10, it is Fully available and scores 1.

Third, Modularity of Availability has four qualitative measurements for each considered parameter. Two of the mappings belong to qualitative measurements of full compliance or Feature Availability to 1, which means the feature/function is seamlessly available. If a feature/function is not available/offered in LMS, then the rubric is set to No Availability as 0. Whereas Mostly to 0.66 reinforces the logic that Features Availability via Plugin, and Availability via Program Script to 0.33 reinforces the logic of Partially. Feature Availability via Plugin means that a feature/function is not seamlessly available in LMS but can be added via a third-party plugin tool by installing it. Lastly, Availability via Program Script means it is neither available by default nor via any plugin, so a program script in LMS is needed to make that feature available.

This scoring function brings quantitative values to [0, 1] interval so these features’ score values can be compatible with the rest of the features/functions. Consider an LMS L for which we need to compute the suitability score, LS, based on its functionality. As mentioned above, the proposed framework categorizes the evaluation criterion into three rubric types. However, every feature/function parameter is grouped in one block while computing the score. This paper maps the qualitative measure to the quantitative score for each parameter in Table 3. For defining the score of an LMS L against a feature/function parameter ‘i’ as LS (i).

By default, each feature/function parameter ‘i’ carries weight 1, i.e. ω(i) = 1. The score for parameter ‘i’ is computed by multiplying the weight ω(i) with the score of the parameter L (i) S, for the LMS L. Now, to compute the overall suitability score LS for an LMS L, we define a simple score aggregation function; as most research papers such as [73] use scoring function; as in the following Eqs 1 and 2:

$$L_{s}=\sum_{i=0}^{n}\omega^{\left(i\right)}.L_{s}^{\left(i\right)}$$
Eq 1 –Unbounded Suitability Score of an LMS Feature

Where ‘***n***’ is the total number of features/functions for the LMS evaluation framework. In this paper, we have more than 400 features/functions. ***L***_***S***_ gives us the suitability score for LMS ***L*** as the best LMS. Hence, the above-mentioned scoring function and discussion in the previous sections help us compute the score for all LMSs. The LMS with a maximum suitability score turns out to be the most suitable.

The suitability score is further processed by dividing the obtained score and the sum of weights of all parameters, which helps to bound the overall suitability score in the [0, 1] interval. This bounded or normalized score, with the default weight settings, implicitly reflects the overall percentage of conformance of LMS to the proposed framework, i.e., a 0.79 score reflects 79% conformance to the defined framework. Similarly, if the difference between two or more parameters is 0.23, it should be treated as 23% less conformance. On the other hand, an unbounded score has the advantage in that it reflects the differences in higher quantitative terms, i.e., it is not bounded to the [0, 1] range. But it fails to show the level of conformance to the required proposed framework of this paper. We leave it to the reader/user to choose any of the two score variants. The bounded scoring function is to be calculated as the following equation:

Ls′=Ls∑i=0nω(i)
Eq 2—Bounded Suitability Score of an LMS Feature

The following sections will cover the evaluation for the scoring function of LMS’s features and functions.

## 4. Features and evaluation of framework on LMS

This section discusses the features and evaluation of different LMSs, which are defined by comparing its features and functions for a scoring function. First, this study focused on the Berkeley evaluation framework, McGill evaluation tool, and previous literature to identify the features, LMS (Learning Management Systems), and standards followed in these studies. Our goal was to propose a framework that is more organized, with additional features, functions, and rubrics. To achieve a well-organized framework, we divided the features into seven departments, each with sub-features and functions. We standardized the framework by adhering to LOM (Learning Object Metadata) standards across various features and functions, informed by extensive literature reviews.

After thorough analysis, we categorized these features into three main rubric types, as shown in Table 2. These types are briefly discussed below:

1. **Binary Rubric**: Features and functions are either available or not, represented by a binary score. Compliance or availability is mapped to 1, and non-compliance or unavailability is mapped to 0.
2. **Analytic Rubric**: Some features have separate scoring criteria with a numerical range. For instance, if a single language is available in an LMS’s multiple languages feature, the score is 0. If the number of available languages ranges from 1 to 5, it is partially (0.33) available. If the range is 5 to 10, it is mostly (0.66) available, and if it exceeds 10, it is fully available, scoring 1.
3. **Modularity of Availability**: This rubric uses four scoring options for each parameter. Full compliance or feature availability is scored as 1, meaning the feature/function is seamlessly available. No availability is scored as 0. Features available via a plugin are scored as 0.66, and features available via a program script are scored as 0.33.

The suitability score is further processed by dividing the obtained score and the sum of weights of all parameters, which helps to bound the overall suitability score in the [0, 1] interval or it is not bounded to the [0, 1] range.

We evaluated the four well known LMSs including, Moodle, Blackboard, TalentLM and canvas to compare their rubrics and performance on the bases of rubrics and suitability as given in Table 4.Lastly, an overview of the evaluation will briefly discuss which scoring rubric impacts the scoring criterion.

**Table 4:** Evaluation of selected LMSs for Un-bounded and bounded scores.

#	Features	Sub-Features and Functions	Un-bounded Score $L_{s}=\sum_{i=0}^{n}\omega^{\left(i\right)}.L_{s}^{\left(i\right)}$				Bounded Score Ls′=Ls∑i=0nω(i)			
			Moodle	Blackboard	TalentLMS	Canvas	Moodle	Blackboard	TalentLMS	Canvas
1	**Platform and Architecture**	Evaluation Score for Interface, Platform & Structure	**28.97**	**28.65**	**28.98**	**30**	**0.93**	**0.92**	**0.93**	**0.97**
2		Evaluation for Vendor’s Architecture	**26.31**	**28**	**28**	**28**	**0.85**	**0.90**	**0.90**	**0.90**
3		Evaluation for Vendor’s Credentials	**4**	**4**	**3.33**	**3.33**	**1**	**1**	**0.83**	**0.83**
4	**Administration and Reporting**	Evaluation for Administration/Site Management	**40.64**	**40.64**	**42**	**42**	**0.97**	**0.97**	**1**	**1**
5		Evaluation for Security	**13.98**	**14.32**	**13.65**	**15**	**0.93**	**0.95**	**0.91**	**1**
6		Evaluation for Reporting	**27.66**	**28**	**28**	**28**	**0.98**	**1**	**1**	**1**
7	**Academic Support Features**	Evaluation for Talent Development and Certificate Tracking	**1.98**	**3**	**3**	**3**	**0.66**	**1**	**1**	**1**
8		Evaluation for Workflow & Approval Process	**4.66**	**4.66**	**4.66**	**4.66**	**0.93**	**0.93**	**0.93**	**0.93**
9		Evaluation for Instructor-led courses	**5.3**	**5.98**	**5.98**	**5.98**	**0.76**	**0.85**	**0.85**	**0.85**
10		Evaluation for Evaluation Feature in LMS	**22**	**22**	**22**	**22**	**1**	**1**	**1**	**1**
11	**Content Development and Management**	Evaluation for Learners’ Feature	**23.65**	**24.99**	**24.99**	**26.33**	**0.94**	**0.96**	**0.96**	**0.99**
12		Evaluation for Learning Content Management	**58.61**	**57.59**	**59.62**	**62**	**0.94**	**0.93**	**0.96**	**1**
13		Evaluation for Learning Content E-library	**2.98**	**4**	**4**	**4**	**0.74**	**1**	**1**	**1**
14		Evaluation for Catalog & Search Feature	**5.66**	**5.66**	**5.66**	**5.66**	**0.94**	**0.94**	**0.94**	**0.94**
15		Evaluation for Content Plagiarism	**1.98**	**1.98**	**1.98**	**1.98**	**0.66**	**0.66**	**0.66**	**0.66**
16	**Smart Features**	Evaluation for Mobile Features	**12.66**	**12.66**	**12.66**	**13**	**0.97**	**0.97**	**0.97**	**1**
17		Evaluation for Gamification	**10.56**	**10.56**	**16**	**16**	**0.66**	**0.66**	**0.94**	**0.94**
18		Evaluation for Virtual Classroom	**9.9**	**9.9**	**13.3**	**15**	**0.66**	**0.66**	**0.87**	**1**
19	**Learners’ Support Features**	Evaluation for Administration of Learner Management	**14.98**	**14.64**	**16**	**16**	**0.94**	**0.91**	**1**	**1**
20		Evaluation for Social Media Features	**20.28**	**22.32**	**22.66**	**23**	**0.81**	**0.89**	**0.91**	**0.92**
21		Evaluation for Communication & Collaboration Features	**17.97**	**20**	**20**	**20**	**0.90**	**1**	**1**	**1**
22	**LMS Support Tools**	Evaluation for LMS Plugins	**7**	**7**	**7**	**7**	**1**	**1**	**1**	**1**
23		Evaluation for E-commerce Features	**3.3**	**5**	**5**	**5**	**0.66**	**1**	**1**	**1**
24		Evaluation for Technical Support	**4**	**4**	**4**	**4**	**1**	**1**	**1**	**1**

### 4.1. Platform and architecture

This feature and function department discusses and evaluates the interface of LMS, technical structure, platform, the architecture of the system, LMS’s vendor’s integration capabilities, and its credentials. The interface, platform & structure discuss the interface of LM systems in terms of its multiple language presentation features, the system’s modularity, and the structure of LMS. The vendor’s architecture & integration section discusses the LMS provider’s ability to integrate with other support software tools and its licensing type. Lastly, the vendor’s credentials are highlighted to check whether the LMS provider has support in Pakistan and how long it has been in the LM system service business. Besides, this section aims to evaluate LM systems’ interface for user-friendliness, compatibility, and customization, integration availability with learning support systems, LM system’s architecture, and other miscellaneous features.

#### 4.1.1. Interface, platform & structure

This section discusses and evaluates the interface of LMS, its platform, and structure. The score focuses on multiple language availability, smartphones view compatibility, availability of integration with ERP, customization availability, and other miscellaneous features. The compatibility and integration of LM systems with other support tools make LMS a technological advantage in a learning environment. Features and functions discussing customization of the LMS interface, branding of institutional colors/logo, and navigation are also presented in the proceeding section, along with its scoring at the end. Appendix A, Table 1 in S1 File discusses the features/functions and scoring for the stated sub-department. Un-bounded and bounded evaluation scores are given in Table 4 and detail is given in Appendix A, Table 1 in S1 File.

This section determines the evaluation score of LM systems’ features and finds a slight advantage with TalentLMS, which has a better integration module with the ERP system. Though Moodle and Blackboard can come to their competition with additional resource deployment. Appendix A, Table 1 in S1 File shows the evaluation scores of the interface, platform, and structure of LMS:

#### 4.1.2. Vendor’s architecture & integration

Selecting an LMS can be tricky if its client-to-be does not fully understand the vendor’s architecture and integration. This section discusses the ability to integrate 3^rd^ party software tools, including courseware tools, administration, and other LM tools. LMS customization tools and their ability to integrate without LMS vendor approval, whereas LM systems’ vendor’s compliance with SCORMs is a feature discussed in the evaluation section at the end of the following table. Appendix A, Table 2 in S1 File discusses the feature, function set, and scoring for the vendor’s architecture and integration. Un-bounded and bounded evaluation scores are given in Table 4 and detail is given in the Appendix A, Table 2 in S1 File.

Appendix A, Table 2 in S1 File discusses the evaluation of the feature and function set for the vendor’s architecture and integration. The evaluation score for features and functions such as different types of database encryption and database support shows the flexibility of availability. However, Moodle LMS can be put up to the mark by installing a plugin or program script customization.

#### 4.1.3. Vendor’s credentials

This section discusses features focusing on the credentials of LMS’s vendor. The vendor’s credentials are matched by measuring the global reach of LMS, its technical support in Pakistan, and tracking capabilities to track licensing and certifications. Lastly, a piece of basic information requires knowing how long an LMS vendor has been in the business. The features and functions are given in Appendix A Table 3 in S1 File. Un-bounded and bounded evaluation scores are given in Table 4 and detail is given in Appendix A, Table 3 in S1 File.

This section evaluates vendors’ credentials as if it has a good number of customers or not, its local support, and business experience. Appendix A, Table 3 in S1 File shows that TalentLMS is new in the LMS arena for the features concerning vendor credentials.

### 4.2. Administration and reporting

The features and functions department discusses the administrative tools to manage the LMS site, which discusses the creation and management of course categories/departments and its evaluation score. The ability of system administrators to create, edit users, and assign special rights to specific users. LM system’s ability to manage site backups and logs. Its security features and functions discuss user authentication available via server-level as well as network-level. Single sign-on (SSO) ability of LMS, enforcement of strong passwords, and external authentication. This section evaluates administration and reporting’s features and functions, such as site administration and administrative module features, learning activity tracking, and other miscellaneous features. It also evaluates security features and functions such as authentication of different user levels, SSO availability, and various features. Lastly, the reporting features and functions include tracking LMS tools, users, activity logs, and activities on the LMS site, along with the support reports.

#### 4.2.1. Administration/site management

These features and functions are set to discuss the availability of separate administrative modules to administer the LMS site. Administration can assign customized rights, creation of learning departments/sub-departments, and their management over the site. The ability of LMS to create and manage site backups. The tracking ability of LMS users by site administration, scaling of LMS user’s traffic, adding and editing, and restoring the ability of LMS and other miscellaneous features and functions. The detailed features and functions are given in Appendix B, Table 4 in S1 File. Un-bounded and bounded evaluation scores are given in Table 4 and detail is given in Appendix B in S1 File.

The score evaluates the site administration and management for different LM systems. Features and functions such as archiving and restoration of users/courses can be achieved for struggling LM systems by the installation of a plugin. Appendix B, Table 4 in S1 File shows the unbounded and bounded scores after the evaluation of LMSs.

#### 4.2.2. Security

This features section is quite important as all transactions of learning resources from LMS personnel/users depend on a secure environment. User authentication via username and password is a basic feature to authenticate a user on the LMS site. Table 4 discusses it with the addition of SSO functionality and external authentication feature. LM system’s ability to enforce strong passwords as well as to force LMS users to change passwords. An internet browser lockdown ability of LMS during LMS-based online examination. These security features and functions enable the administration of LM Systems in a secured online environment.

The above section evaluates the core features and functions of the security in which the evaluation score of LM systems varies due to the unavailability of built-in features and functions of SSO and external authentication. Such features and functions can be added via plugin or program script customization. Un-bounded and bounded evaluation scores are given in Table 4 and detail is given in Appendix B, Table 5 in S1 File.

#### 4.2.3. Reporting features

This section discusses features and functions to monitor each LMS resource and later manage it administratively. Reporting features help administrators track any LMS record that can be of any learning content, resource, or both. These features and functions include tracking LMS tools and users record. Types of reporting to support management and administration of LMS are drill-down and roll-up reports. An automated report of LMS to track/monitor task-specific transactions, whereas customized reports and out-of-the-box reporting features also make these features an important category. The features and functions are given in Table 6.

This section evaluates different LMS through scoring of features and function of reporting section. Features and functions like out-of-the-box reports and reporting formats can be added to struggling LM systems via plugin or programming script customization. Un-bounded and bounded evaluation scores are given in Table 4 and detail is given in Appendix B, Table 6 in S1 File.

### 4.3. Academic support features

This section discusses features, functions, and evaluations supporting academic proceedings, such as tracking and managing learners’ course certificates, approval, content learners’ career, and competency management tools. The workflow and approval process features and functions table covers the management of waitlists and learners’ registration options for an LMS course. Features required for instructor-led courses that cover conflict management of instructor-led class schedules are also discussed. Lastly, the evaluation table represents an evaluation of learning resources and activities attempted/performed by learners in a course. Also, it categorizes if LM systems can restrict an exam to an IP address scheme only for security reasons. These activities might be assignments, quizzes, or miscellaneous. It also covers the grading tools that help to evaluate learners’ activities proactively.

#### 4.3.1. Talent development & certificate tracking

This section covers features and functions that track learners’ course/career in the LMS environment, learners’ certification management, and competency throughout LMS, which are given in Appendix C, Table 7 in S1 File.

Table 7 shows the scoring, which evaluates certificate tracking and its management tools. Moodle does not have a built-in tool for the feature, but it can support these features via plugin installation. Un-bounded and bounded evaluation scores are given in Table 4 and detail is given in Appendix C, Table 7 in S1 File.

#### 4.3.2. Workflow & approval process

This section covers features and functions of workflow and approval process in LMS, including the management of a waitlist so that a teacher could allow or deny a learner to enroll in an LMS course. It also includes a self-enrollment option to enable a learner to enroll automatically. Registration of a learner with a course manager’s approval is different than a waitlist/queue, which manages enrollment requests. Lastly, the course registration confirmation email is a few features in Appendix C, Table 8 in S1 File.

Table 8 present the score to evaluate the workflow and approval process, which evaluates features such as managing a waitlist queue, self/automatic registration option for learners, and notification via email. Management of the registration waitlist of learners can be managed by adding an external plugin in LM systems. Un-bounded and bounded evaluation scores are given in Table 4 and detail is given in Appendix C, Table 8 in S1 File.

#### 4.3.3. Instructor-led courses

This section focuses on institutional courses taught in a classroom environment through the features table. These features cover functions that course administrators can assign in LMS as instructor-led courses. The ability of such courses to be tracked throughout LMS, conflict/clash checking between class schedules and learners’ course schedules. The ability of LM systems to limit learners in an instructor-led course and to reserve classrooms. The features and functions are given in Appendix C, Table 9 in S1 File.

The above scores features and functions comprise instructor-led course assigning, tracking, limiting learners’ strength in ILT courses, and miscellaneous features. Instructor-led courses are evaluated from the scoring Table 12 in S1 File, which shows that most features are not available as built-in features but can be added via plugin/add-on installation in LM systems. Un-bounded and bounded evaluation scores are given in Table 4 and detail is given in Appendix C, Table 9 in S1 File.

#### 4.3.4. Evaluation

This section covers features and functions for evaluation which is a critical part of any LMS as every task operated by the learner/student should be evaluated by the instructor/course administrator. The following features and functions are discussed to rate whether that specific LMS has how much capacity to comprehend the evaluation needs of the institute. For example, the following table discusses assessment tools for assignments, quizzes, chats, online discussions, and other learning activities. Moreover, an IP restriction ability check is also represented to restrict LMS course assessment. Lastly, a few features and functions are illustrated in Appendix C, Table 10 in S1 File to evaluate LM systems’ grading functionality.

This section evaluates the features and functions of evaluation tools in LM systems, including assessment tools/features for quizzes, assignments, attendance, and other learning activities. These assessments are core features in the evaluation process of any learner. The evaluation security feature is also included in the table to ensure IP-based restrictions on quizzes/Exams. Un-bounded and bounded evaluation scores are given in Table 4 and detail is given in Appendix C in S1 File. Scoring table covering the most aspects of stated features and functions.

### 4.4. Content development and management

Content development and its management is a very important aspect of any LMS. This department covers learners’ oriented content development tools with support features and functions, which cover course allocation, learners’ view of course contents, ability to create a course online notes, posting feedback on online submissions, and various features with its evaluations. The other section discusses the tools required to manage these features and functions, such as the ability to import and export course content, a built-in course content management HTML, course as a single-activity e.g., quiz/assignment/wiki/etc., course upload tools, course content management tools, online content embedding options for YouTube/etc., and other content management support tools. This section also discusses the e-library and content’s catalog search feature table as a separate table. Lastly, the content plagiarism check feature is also discussed.

#### 4.4.1. Learners’ features

This section discusses learners’ oriented features and functions to access and use learning content. The extensive functionality of learning content w.r.t learners’ front can be measured from this section. Course assigning, learners’ management in LMS courses, administratively concerned learners’ support features, learning activities such as questionnaires, surveys, and other learners’ centered features are covered in this section. The details of features and functions are given in Appendix D, Table 11 in S1 File.

Scoring Table 11 in S1 File evaluates learners’ features and functions that focus on learners’ oriented features and functions. Features and functions such as properly structured LMS notifications which Moodle and Blackboard struggle to have seamlessly, can be overcome by an add-on/plugin installation. Similarly, other features like course availability view, rating, course registration waiting list, bookmarking of LMS course, and likewise features that score less than one can provide such features seamlessly via add-on/plugin installation. Un-bounded and bounded evaluation scores are given in Table 4 and detail is given in Appendix D, Table 11 in S1 File.

#### 4.4.2. Learning content management

Course administrators or instructors can manage learning content on the LMS site. These features and functions include importing and exporting course data into/from LMS. A built-in feature for the HTML editor is an important tool to customize learning content shared in the LMS course, which is also discussed in the features table. Courses such as Wikis, quizzes, assignments, and surveys are also discussed as a single learning activity. The learning content management section discusses these features and functions given in Appendix D, Table 12 in S1 File.

The scoring Table 12 in S1 File evaluates features and functions for learning content management tools. Features such as course as a single activity, LMS course linking to other online learning systems, instructor-learner learning mashups, virtual learning environments, and likewise features that are not available seamlessly in LM systems can be added via programming script or plugin/add-on installation. Un-bounded and bounded evaluation scores are given in Table 4 and detail is given in Appendix D, Table 12 in S1 File.

#### 4.4.3. Learning content e-library

An E-library is a handy tool for learners and instructors in the LMS environment. This section discusses e-library features and functions in LMS, such as a content library that can be uploaded for learning reference purposes. Research paper management and wiki tool availability are also kept in the features table. These features are given in Appendix D, Table 13 in S1 File.

Features and functions for learning content e-library are evaluated in Table 13 in S1 File. Features such as reference tagging, learning vocabulary/glossary, and managing research articles are features that Moodle provides with only plugin installation. Un-bounded and bounded evaluation scores are given in Table 4 and detail is given in Appendix D, Table 13 in S1 File.

#### 4.4.4. Catalog & search feature

Cataloging learning content throughout LMS helps to ease navigation for LMS users. Moreover, the search feature in LMS helps a user to find respective learning content easily. It is important if the search feature is clear to use, returns results in easy-to-understand review results, and content tagging by learners. Features and functions for cataloging learning content and its search are featured in Appendix D, Table 14 in S1 File.

Table 14 in S1 File presents the scoring for catalog and search feature evaluation. Features and functions regarding advanced search options for learning content with custom fields can be added via add-on/plugin installation. Un-bounded and bounded evaluation scores are given in Table 4 and detail is given in Appendix D, Table 14 in S1 File.

#### 4.4.5. Content plagiarism

It is the most needed tool to check any plagiarized content submitted by learners. That content may be an assignment, research article, or quiz submission. Appendix D, Table 15 in S1 File also contains plagiarism checking of different content types i.e., programming and equations.

Scoring Table 15 in S1 File evaluates the content plagiarism of different plagiarism tools available in LM systems through third-party software that can be added into LMS via an add-on/plugin. Un-bounded and bounded evaluation scores are given in Table 4 and detail is given in Appendix D, Table 15 in S1 File.

### 4.5. Smart features

This department contains smart features of LMS that distinguish it from traditional learning environments. These smart features and functions give LMS a technological productivity edge over other content management solutions. These smart features are mobile features that cover learning management-oriented mobile/smartphone features and functions. These functions include administrative tools to manage LMS sites via smartphone and content reading/submission capabilities for learners on mobile. This section discusses Gamification, a top-rated feature among modern learning management systems. Gamification tool’s features are generally discussed here, which covers almost all categories of Gamification. Lastly, virtual classroom features and functions are discussed.

#### 4.5.1. Mobile features

Mobile devices, especially smartphones, are quite famous among the modern generation. Therefore, LMS must have mobile-friendly features and functions so it can be accessed easily from mobile devices. These features and functions include the ability to access course contents and manage them by instructors, course administrators, and site administration, along with its evaluation. Learners’ management tools include adding/editing LMS users and reporting features through a mobile device. Lastly, a smartphone mobile app or LM system. Mobile features and functions are discussed in Appendix E, Table 16 in S1 File.

Table 16 in S1 File presents the scoring for features and functions that will help evaluate mobile/smartphone features that focus on sending a text message and can be added via third-party software or installed via an add-on/plugin. Un-bounded and bounded evaluation scores are given in Table 4 and detail is given in Appendix E, Table 16 in S1 File.

#### 4.5.2. Gamification

Gamification is a great tool to mix enjoyment, motivation, and the flow of creative engagement with learning activities and resources. Learning management systems have this tool to create a competitive environment among learners to motivate learners of LM systems towards a greater learning-oriented goal. Features of Gamification given in Appendix E, Table 17 in S1 File include functions such as achievement badges, leaderboards of learners concerning performance, audio and video effects including other visual aid animation tools, users’ profiles, virtual goods, etc.

Table 17 in S1 File presents the scoring for features focusing on Gamification functions in Moodle and Blackboard, which are available via add-on/plugin. Un-bounded and bounded evaluation scores are given in Table 4 and detail is given in Appendix E, Table 17 in S1 File.

#### 4.5.3. Virtual classroom

It’s a tool that gives virtual reality to a classroom environment. Learner/student is usually not in the classroom but rather behind the computer screen, taking full participation in the classroom session, raising questions, answering pop-up questions from the instructor, and participating in video/audio/text group discussions. These features include creating a virtual classroom, disabling it, enrollment of learners, video conferencing, audio calls, chat room availability, and other features facilitating virtual classroom features in the LM system. Appendix A, Table 18 in S1 File has the features and functions of the virtual classroom for LMS.

In a virtual classroom environment, a student is usually not in the classroom but rather behind the computer screen, taking full participation in the classroom session, raising questions, answering pop-up questions from the instructor, and participating in video/audio/text group discussions. An evaluated score of features meeting the ‘0.66’ score can be added via add-on/plugin. Table 18 in S1 File evaluates these features and functions. Un-bounded and bounded evaluation scores are given in Table 4 and detail is given in Appendix E, Table 18 in S1 File.

### 4.6. Learners’ support features

This department of features and functions covers learners’ focused support tools and evaluations. These features and functions include administration of learners’ management which discusses course registration management, attendance module, course assigning options by a teacher to other LMS users, bulk actions in LMS for adding bulk users and courses, and other learners, management options. The social media feature table represents many functions, such as integrating LMS with social media engines, e.g., Facebook, Twitter, etc., and other socially interactive support features in the LM system. Lastly, communication & collaboration features help a learner connect and get informed through email notifications on several learning tools.

#### 4.6.1. Administration of learner management

This sub-department covers features administrating learners’ access, manual enrollment, bulk actions from the course and site administrators, activating/enabling and deactivating/suspending LM system’s accounts, calendar functions to save upcoming learning activity from notifying alerts, and miscellaneous functions. In Appendix F, Table 19 in S1 File features these functions.

Table 19 in S1 File presents the scoring of course registration, course enrollments, adding/editing of learners in LMS, etc., course registration management feature, and instructor’s schedule management feature under the administration of learner management functions. Other features not seamlessly (score equal to ‘1’) available can be made available in the LM system by installing an add-on/plugin. Un-bounded and bounded evaluation scores are given in Table 4 and detail is given in Appendix F, Table 19 in S1 File.

#### 4.6.2. Social media features

Social media is a tool to connect and create a social environment in LMS. This environment is helpful in learners’ information and knowledge sharing. Such features and functions are the ability of LMS to integrate with Facebook, Twitter, and YouTube. A secure social media environment is also integral to this features table. The following table shows other features and functions to support LMS’s social media features. Appendix F, Table 20 in S1 File features social media functions in LMS.

The functions and features of an LM system that connects learners and instructors on a social media platform are an important need in a modern learning environment. Social media features are evaluated in the this section and in the Table 20 in S1 File. Un-bounded and bounded evaluation scores are given in Table 4 and detail is given in Appendix F, Table 20 in S1 File.

#### 4.6.3. Communication & collaboration features

A system must provide such features and functions for LMS users to connect, communicate and collaborate in an LMS environment. For example, notifications via email and SMS through LMS, calendar management in LMS, messaging feature, and other features and functions as mentioned in Appendix F, Table 21 in S1 File.

Table 21 in S1 File shows the score to evaluate communication and collaboration features focusing on communication tools within LMS and its management. Un-bounded and bounded evaluation scores are given in Table 4 and detail is given in Appendix F, Table 21 in S1 File.

### 4.7. LMS support tools

This features department in LMS discusses advanced features in LM systems that differentiate many LM systems along with the evaluations. Moreover, these features and functions urge the importance of LMS among learning-oriented institutes. Once LMS is set up and configured, discussed features and functions will allow us to make advancements towards a better LMS environment. This includes the LMS plugins feature, which represents the availability of add-ons that can add additional support/technical tools such as themes, administration, assessments, and others. Later, a table featuring e-commerce functions is represented, including purchasing and other e-commerce transaction tools. Lastly, technical support features involving online and offline technical support tools are described.

#### 4.7.1. LMS plugins

Plugins features enable LMS to advance in different areas in LMS functionality, such as administrative tools, security, themes, etc., as given in Appendix G, Table 22 in S1 File.

Table 22 in S1 File presents the scoring of Plugin/add-ons add value by enabling customization in LMS in themes, administration tools, and other functional/non-functional tools to evaluate the plugin directory. Un-bounded and bounded evaluation scores are given in Table 4 and detail is given in Appendix G, Table 22 in S1 File.

#### 4.7.2. E-commerce features

E-commerce in LMS is a tool to buy learning content, just like paying to register for a traditional classroom course. This e-commerce feature has to be comprising the following features and functions in Appendix G Table 23 in S1 File. The same table presents the scoring for e-commerce features that evaluate payment procedures and its management in LMS. Un-bounded and bounded evaluation scores are given in Table 4 and detail is given in Appendix G, Table 23 in S1 File.

#### 4.7.3. Technical support

Any technical issue can be addressed by LMS administrators that are provided for LMS users. Moreover, the vendor expects to give technical support to site administrators if needed. Further details and its scoring are given in Appendix G, Table 24 in S1 File to evaluate technical support features in LMS. Un-bounded and bounded evaluation scores are given in Table 4 and detail is given in Appendix G, Table 24 in S1 File.

We have included a summary of the data in the Appendixes (A–G) in S1 File within the supplementary material collected during the trial of LMSs. It will help readers to understand the practical application and verify the effectiveness of our framework.

## 5. Discussion of overview of LM systems’ scoring rubric table

This section will briefly overview the evaluation of LM systems’ features and functions concerning evaluation tables. Table 5 shows the number of rubric descriptions vs. respective rubric types:

**Table 5:** Rubric type vs. No. of rubrics description.

Rubric Type	No. of Rubric Description of Respective Type
*Binary Rubric*	*168*
*Modularity of Availability*	*240*
*Analytic Rubric*	*4*
*Total*	*412*

### 5.1. Overview of LM systems’ scoring for collective rubric tables

Evaluation overview for all features’ department table is overviewed in Tables 6 and 7 along with the unbonded and bounded score, which is given below:

**Table 6:** Overview for all features’ department for selected LMSs.

Features’ Department	Sub-Features Department	Moodle	Blackboard	TalentLMS	Canvas
**Platform and Architecture**	Interface, Platform & Structure	0.93	0.92	0.93	0.97
	Vendor’s Architecture & Integration	0.85	0.90	0.90	0.90
	Vendor’s Credentials	1	1	0.83	0.83
**Administration and Reporting**	Administration/Site Management	0.97	0.97	1	1
	Security	0.93	0.95	0.91	1
	Reporting Features	0.98	1	1	1
**Academic Support Features**	Talent Development & Certificate Tracking	0.66	1	1	1
	Workflow & Approval Process	0.93	0.93	0.93	0.93
	Instructor-led courses	0.76	0.85	0.85	0.85
	Evaluation	1	1	1	1
**Content Development and Management**	Learners’ Features	0.94	0.96	0.96	0.99
	Learning Content Management	0.94	0.93	0.96	1
	Learning Content E-Library	0.74	1	1	1
	Catalog & Search Feature	0.94	0.94	0.94	0.94
	Content Plagiarism	0.66	0.66	0.66	0.66
**Smart Features**	Mobile Features	0.97	0.97	0.97	1
	Gamification	0.66	0.66	0.94	0.94
	Virtual Classroom	0.66	0.66	0.87	1
**Learners’ Support Features**	Administration of Learner Management	0.94	0.91	1	1
	Social Media Features	0.81	0.89	0.91	0.92
	Communication & Collaboration Features	0.90	1	1	1
**LMS Support Tools**	LMS Plugins	1	1	1	1
	E-commerce Features	0.66	1	1	1
	Technical Support	1	1	1	1
*Total*: **7**	*Total*: **24**				

**Table 7:** Overview for an unbounded and bounded score of all features’ department.

Features’ Department	Un-bounded		$L_{s}=\sum_{i=0}^{n}\omega^{\left(i\right)}.L_{s}^{\left(i\right)}$			Bounded		Ls′=Ls∑i=0nω(i)	
	Moodle	Blackboard		TalentLMS	Canvas	Moodle	Blackboard	TalentLMS	Canvas
Platform and Architecture	2.78	2.82		2.66	2.7	0.93	0.94	0.89	0.90
Administration and Reporting	2.88	2.92		2.91	3	0.96	0.97	0.97	1
Academic Support Features	3.35	3.78		3.78	3.78	0.84	0.94	0.94	0.94
Content Development and Management	4.22	4.49		4.52	4.59	0.84	0.90	0.90	0.92
Smart Features	2.29	2.29		2.78	2.94	0.76	0.76	0.93	0.98
Learners’ Support Features	2.65	2.8		2.91	2.92	0.88	0.93	0.97	0.97
LMS Support Tools	2.66	3		3	3	0.88	1	1	1
*Total*: **7**									

## 6. Concluding evaluation of LM systems’ scoring rubric tables

Table 8 concludes the overall discussion of the evaluation of LMS scoring tables:

**Table 8:** Overview evaluation for overall features and functions categories.

#	Features’ Main Categories	Moodle	Blackboard	TalentLMS	Canvas
1	Platform and Architecture	0.93	0.94	0.89	0.90
2	Administration and Reporting	0.96	0.970	0.97	1
3	Academic Support Features	0.84	0.94	0.94	0.94
4	Content Development and Management	0.84	0.90	0.90	0.92
5	Smart Features	0.76	0.76	0.93	0.98
6	Learners’ Support Features	0.88	0.93	0.97	0.97
7	LMS Support Tools	0.88	1	1	1
1	**Un-bounded Score** $L_{s}=\sum_{i=0}^{n}\omega^{\left(i\right)}.L_{s}^{\left(i\right)}$	**6.09**	**6.44**	**6.6**	**6.71**
2	**Bounded Score** Ls′=Ls∑i=0nω(i)	**0.87**	**0.92**	**0.94**	**0.96**

**Fig 8:**
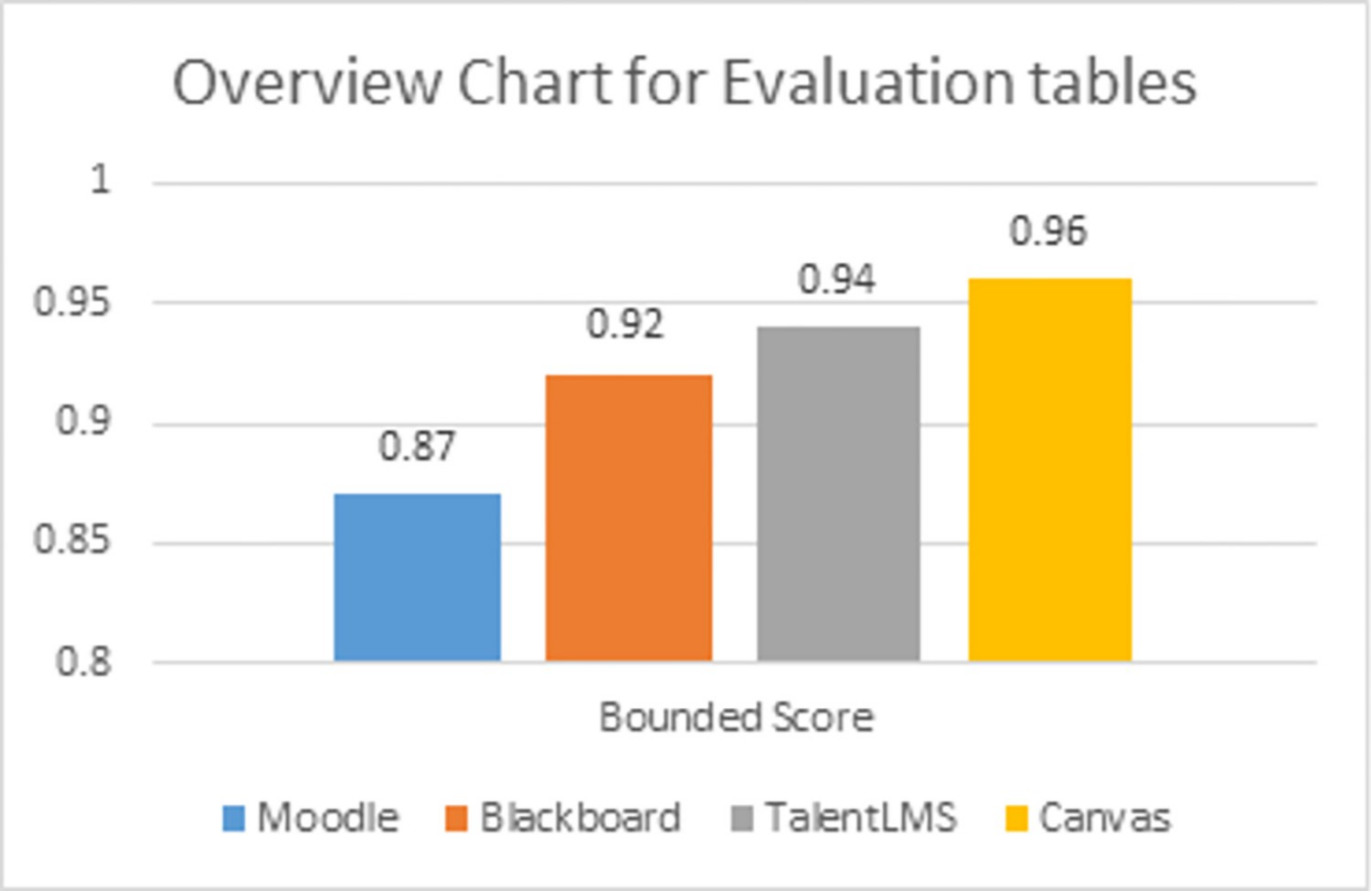
Comparison and evaluation of selected LMSs.

Concluding the discussion and analysis of evaluation for learning management systems’ features and functions, LM systems’ evaluation score is shown in Fig 8. From the above overview evaluation, tables show Canvas has covered more functions and features according to FRSF, giving each feature a fair weightage of 1.

Some detailed case studies or practical examples of how different features of Learning Management Systems (LMSs) impact user experience:

A large university implemented an LMS that offered extensive customization and branding capabilities to align with the institution’s identity. These customization options made users feel more at home, resulting in higher engagement rates and a more intuitive interaction with the system. This, in turn, led to better adoption rates and a smoother learning experience.

Similarly, a corporate training program adopted an LMS with a robust mobile app, enabling employees to access training modules on their smartphones and tablets. The mobile accessibility allowed users to interact with the LMS from any location, at any time, making the learning process more convenient and adaptable to individual schedules. This increased course completion rates and overall user satisfaction.

In another example, a healthcare training provider utilized an LMS equipped with advanced analytics and reporting features. These tools allowed instructors to monitor learner progress in real-time, identify areas where students were struggling, and provide targeted support. Access to detailed analytics enabled instructors to tailor the learning experience to individual needs, offering timely interventions and support. This resulted in a more personalized and effective educational journey for learners, increasing their chances of success.

A tech company, on the other hand, implemented an LMS featuring built-in social learning tools such as discussion forums, group workspaces, and peer review systems. These social learning and collaboration tools enhanced the sense of community and encouraged peer-to-peer interaction, leading to deeper learning and a more engaging experience. This fostered a collaborative environment where learners felt supported and motivated to participate.

By incorporating these real-world examples, we aim to provide deeper insights into how different LMS features impact user experience. Our goal is to make the comparison more relevant and engaging for readers, ultimately offering a more comprehensive understanding of the practical implications of the findings.

## 7. Conclusion and future directions

LMS is a leading learning tool not confined to higher education, schools, colleges, and corporate training. So, the future holds a major impact of LMS on the education and corporate sector. This study has proposed a scoring function to evaluate different LMS concerning various features and functions. These features and functions are also valuable in terms of IEEE LOM standards that discuss seven categories of features with three to five subcategories of features and functions. These subcategories have relevant components and functions concerning their respective heading. Rubric description for the corresponding feature evaluates and sets the score for the respective LMS solution. Each score is recorded and scored for every subcategory. Each subcategory score is summed up and recorded for its respective category, both as bounded and unbounded scores. Lastly, these scores are compared to record the best available choice for learning management solutions in the market.

As per the discussion in our previous section about the projected market worth of LMS and its global impact, LMS needs to be ready for the challenges it may face. Let’s bring security features and functions into account, it is important to be accounted for and up to the mark. Secondly, an LM systems solution provider needs to implement more additional widgets/plugins/add-ons so that different external tools can be used to facilitate learners and teachers. Thirdly, adding portable learning components in an LMS testing environment must be available so that different institutes can carry out different features and functions according to their educational needs/academic environments. Fourthly, as LMS has become more advanced as a cloud-based solution. So as much as F2F interaction is essential in (Instructor-led training) ILT courses at campuses, a supportive instructional LMS must be met in accordance with the knowledge of instruction and faculty’s understanding. The fifth future direction of this study would be to suggest an interoperable and integrate-able friendly learning environment to leverage analytical applications. The sixth future direction is that an LMS must be more collaborative and integrated in terms of user experience so that teachers would make LMS features an integral part of their course, bringing us to the next point. The seventh future direction is the best utilization of LMS must be achieved so that both teachers and students meet their expectations and standard of use. LMS must be optimize-able to enhance teachers’ and students’ experience. The eighth future direction is to have better-skilled teachers and students for LMS usage through better LMS training, support, and a better intuitive interface. It means that an intuitive interface for maximizing LMS productivity and an individualized assessment for calibration of training tools with users’ needs are few of the many on the list. Smartphones have become a necessity and proven to be a positive tool for learning technology, so the ninth future direction would be to optimize a 24/7 engagement, mobile-friendly LMS environment. This study’s tenth and last future direction suggest a personalized LMS interface that adds value to LMS by promoting/optimizing success strategies within and across courses.

## Supporting information

S1 File (DOCX)
