# Supplementary material for: An evaluation framework and comparative analysis of the widely used learning management systems
Source: PLoS One. 2024 Dec 19;19(12):e0311111. doi: 10.1371/journal.pone.0311111 (PMC11658596; doi:10.1371/journal.pone.0311111)
Supplement: S1 File — (DOCX) [file pone.0311111.s001.docx]

**Appendix A**

**Platform and Architecture**

**Table 1 – Evaluation for Interface, Platform & Structure**

| # | Features and Functions | Rubric Description | Moodle | Blackboard | TalentLMS | Canvas |
| --- | --- | --- | --- | --- | --- | --- |
| 1 | LMS has multiple languages | 1<x<6 then partially; 5<x<11 then mostly; x>10 then feature available | 1 | 1 | 1 | 1 |
| 2 | Single learning items can be displayed in multiple languages | 1<x<6 then partially; 5<x<11 then mostly; x>10 then feature available | 1 | 1 | 1 | 1 |
| 3 | LMS website is compatible with mobile devices and tablets view adjustment | Binary Rubric | 1 | 1 | 1 | 1 |
| 4 | LMS is modular by default; every tool of LMS is in a proper heading; there is no mixing/ duplication. | Modularity of Availability | 1 | 1 | 1 | 1 |
| 5 | Users have customization rights to edit additional details in their user interface | Binary Rubric | 1 | 1 | 1 | 1 |
| 6 | Single-sign-on functionality with third-party tools is available | Modularity of Availability | 0.66 | 1 | 1 | 1 |
| 7 | Administrators can modify LMS navigation appearance | Modularity of Availability | 0.66 | 0.66 | 0.66 | 1 |
| 8 | LMS navigation is user-friendly; once a user is in an end zone, he can navigate back to the top | Binary Rubric | 1 | 1 | 1 | 1 |
| 9 | LMS integration with ERP systems is available | Modularity of Availability | 0.33 | 0.33 | 0.66 | 1 |
| 10 | Login page (e.g., logo and institutional colors) customization available for administrators | Modularity of Availability | 0.66 | 0.66 | 0.66 | 1 |
| 11 | In-system to use your branding (e.g., logo and institutional colors) customization | Modularity of Availability | 1 | 1 | 1 | 1 |
| 12 | Branding theme for the Interface is manageable | Binary Rubric | 1 | 1 | 1 | 1 |
| 13 | User theme settings can be managed administratively | Binary Rubric | 1 | 1 | 1 | 1 |
| 14 | Platform's Software Updating: Thin Client features availability | Binary Rubric | 1 | 1 | 1 | 1 |
| 15 | Platform's Hardware Updating: Database movement is swift from one hardware to another | Binary Rubric | 1 | 1 | 1 | 1 |
| 16 | LMS Installation is based on Hosted | Binary Rubric | 1 | 1 | 1 | 1 |
| 17 | LMS theme is manageable for multiple departments | Modularity of Availability | 0.66 | 1 | 1 | 1 |
| 18 | Different course types/formats can be set for multiple departments | Modularity of Availability | 1 | 1 | 1 | 1 |
| 19 | Customizable home page for each user/Learner | Modularity of Availability | 1 | 1 | 1 | 1 |
| 20 | Cloud hosting service is available | Modularity of Availability | 1 | 1 | 1 | 1 |
| 21 | LMS offers a Redundancy plan | Binary Rubric | 1 | 1 | 1 | 1 |
| 22 | The LMS supports Internet Explorer | Binary Rubric | 1 | 1 | 1 | 1 |
| 23 | The LMS supports Firefox | Binary Rubric | 1 | 1 | 1 | 1 |
| 24 | The LMS supports Chrome | Binary Rubric | 1 | 1 | 1 | 1 |
| 25 | The LMS supports Safari | Binary Rubric | 1 | 1 | 1 | 1 |
| 26 | Linking/pairing two LMS via a network is available | Binary Rubric | 1 | 1 | 1 | 1 |
| 27 | Ability to accommodate different time zones for live events | Binary Rubric | 1 | 1 | 1 | 1 |
| 28 | LMS keeps the interface organized and clean for learners | Binary Rubric | 1 | 1 | 1 | 1 |
| 29 | The user interface is customizable through plugins | Binary Rubric | 1 | 1 | 1 | 1 |
| 30 | LMS is an open-source software | Binary Rubric | 1 | 0 | 0 | 0 |
| 31 | LMS database is flexible for import/export in/to LMS | Binary Rubric | 1 | 1 | 1 | 1 |

**Table 2 – Evaluation for Vendor's Architecture**

| # | Features and Functions | Rubric Description | Moodle | Blackboard | TalentLMS | Canvas |
| --- | --- | --- | --- | --- | --- | --- |
| 1 | Ability to comply with 3rd Party Courseware Tools | Binary Rubric | 1 | 1 | 1 | 1 |
| 2 | Ability to comply with 3rd Party Authoring Tools | Binary Rubric | 1 | 1 | 1 | 1 |
| 3 | Ability to comply with 3rd Party Webcasting/Virtual Classroom Tools | Binary Rubric | 1 | 1 | 1 | 1 |
| 4 | Compliance with 3rd Party Teleconferencing Tools | Binary Rubric | 1 | 1 | 1 | 1 |
| 5 | Compliance with SCORM | Binary Rubric | 1 | 1 | 1 | 1 |
| 6 | Customizable Fonts | Binary Rubric | 1 | 1 | 1 | 1 |
| 7 | Customizable Themes | Binary Rubric | 1 | 1 | 1 | 1 |
| 8 | Customizable Functionality Blocks | Binary Rubric | 1 | 1 | 1 | 1 |
| 9 | Customizable Colors | Binary Rubric | 1 | 1 | 1 | 1 |
| 10 | Customizable Dashboard | Binary Rubric | 1 | 1 | 1 | 1 |
| 11 | Legacy System Integration | Binary Rubric | 1 | 1 | 1 | 1 |
| 12 | Licensing is Free | Binary Rubric | 1 | 0 | 0 | 0 |
| 13 | Licensing is Open Source | Binary Rubric | 1 | 0 | 0 | 0 |
| 14 | Licensing is Perpetual | Binary Rubric | 0 | 1 | 1 | 1 |
| 15 | Licensing is Annual | Binary Rubric | 0 | 1 | 1 | 1 |
| 16 | A special software development kit required for LMS customized development | Binary Rubric | 0 | 0 | 0 | 0 |
| 17 | Admins are offered methods of data exchange supported | Binary Rubric | 1 | 1 | 1 | 1 |
| 18 | Admins are offered encryption methods supported | Modularity of Availability | 0.66 | 1 | 1 | 1 |
| 19 | The system can feed into LMS/HRIS/ERP systems (pull and push). | Modularity of Availability | 0.66 | 1 | 1 | 1 |
| 20 | MySQL Database Supported | Modularity of Availability | 1 | 1 | 1 | 1 |
| 21 | ORACLE Database Supported | Modularity of Availability | 0.33 | 1 | 1 | 1 |
| 22 | The ability of a system to support statistical analysis of assessment results | Modularity of Availability | 0.66 | 1 | 1 | 1 |
| 23 | Ability to create multiple institutions | Binary Rubric | 1 | 1 | 1 | 1 |
| 24 | Ability to import/export grades from the LMS using APIs | Binary Rubric | 1 | 1 | 1 | 1 |
| 25 | Ability to integrate Turnitin with the LMS | Binary Rubric | 1 | 1 | 1 | 1 |
| 26 | Ability to integrate library systems with the LMS interface | Binary Rubric | 1 | 1 | 1 | 1 |
| 27 | LMS APIs and web services to link to other systems | Binary Rubric | 1 | 1 | 1 | 1 |
| 28 | Utility to upload SCORM without vendor assistance (single course upload tool) | Binary Rubric | 1 | 1 | 1 | 1 |
| 29 | Utility to upload SCORM without vendor assistance (bulk course upload tool) | Binary Rubric | 1 | 1 | 1 | 1 |
| 30 | Support for hierarchical domains at the institute/enterprise or other defined levels and the ability to spawn new domains without vendor involvement | Binary Rubric | 1 | 1 | 1 | 1 |
| 31 | The system allows configuration of the site navigation easily without vendor involvement | Binary Rubric | 1 | 1 | 1 | 1 |

**Table 3 – Evaluation for Vendor's Credentials**

| # | Features and Functions | Rubric Description | Moodle | Blackboard | TalentLMS | Canvas |
| --- | --- | --- | --- | --- | --- | --- |
| 1 | The global reach for the LMS (number of users, systems and customers, industries)? | 1<x<6 then partially; 5<x<11 then customizable; x>10 then feature available | 1 | 1 | 1 | 1 |
| 2 | The vendor has local support in Pakistan | Binary Rubric | 1 | 1 | 1 | 1 |
| 3 | Track employee licensing and certifications, including expiration dates | Binary Rubric | 1 | 1 | 1 | 1 |
| 4 | How long in business? | less than 5 years=No; less than 8 years=Partially; less than 12 years=Mostly; more than 12 years=Fully | 1 | 1 | 0.33 | 0.33 |

**Appendix B**

**Administration and Reporting**

**Table 4 - Evaluation for Administration/Site Management**

| # | Features and Functions | Rubric Description | Moodle | Blackboard | TalentLMS | Canvas |
| --- | --- | --- | --- | --- | --- | --- |
| 1 | A separate module for administrators is available | Binary Rubric | 1 | 1 | 1 | 1 |
| 2 | System administrators can configure rights/security settings for users on different privileged levels of LMS | Binary Rubric | 1 | 1 | 1 | 1 |
| 3 | Multiple Learning Departments can be created under one LMS | Modularity of Availability | 1 | 1 | 1 | 1 |
| 4 | Different departments' courses can be managed under one LMS | Modularity of Availability | 1 | 1 | 1 | 1 |
| 5 | Different departments' courses can be treated as independent (separate entities w.r.t grading/format) under one LMS | Modularity of Availability | 1 | 1 | 1 | 1 |
| 6 | Customized programming can be added to the application | Modularity of Availability | 1 | 1 | 1 | 1 |
| 7 | The ability to handle backup and retrieval options at the system administrator level is available | Modularity of Availability | 1 | 1 | 1 | 1 |
| 8 | Conversion from a .bak archive into the LMS is easy and thorough | Modularity of Availability | 1 | 1 | 1 | 1 |
| 9 | Maintenance Backups can be taken as per the administrator requirement of any module | Modularity of Availability | 1 | 1 | 1 | 1 |
| 10 | LMS allows Learner Tracking for any course activity | Modularity of Availability | 1 | 1 | 1 | 1 |
| 11 | LMS allows Learner Tracking via IP address | Modularity of Availability | 1 | 1 | 1 | 1 |
| 12 | An administrator manages learners' access controls | Binary Rubric | 1 | 1 | 1 | 1 |
| 13 | An administrator manages instructors' access controls | Binary Rubric | 1 | 1 | 1 | 1 |
| 14 | LMS has a plugins module | Binary Rubric | 1 | 1 | 1 | 1 |
| 15 | Required functionality can be easily added via available plugins | Binary Rubric | 1 | 1 | 1 | 1 |
| 16 | Web-based access to an administrative panel | Binary Rubric | 1 | 1 | 1 | 1 |
| 17 | Web-based editing in administrative panel availability | Binary Rubric | 1 | 1 | 1 | 1 |
| 18 | Create/assign new permissions and roles. | Modularity of Availability | 1 | 1 | 1 | 1 |
| 19 | Edit/Delete new permissions and roles. | Modularity of Availability | 1 | 1 | 1 | 1 |
| 20 | System Admins can edit/delete any role’s announcements. | Binary Rubric | 1 | 1 | 1 | 1 |
| 21 | Detailed Learner's Log-in/out tracking is available | Modularity of Availability | 1 | 1 | 1 | 1 |
| 22 | Detailed Learner's last accessed tracking available | Modularity of Availability | 1 | 1 | 1 | 1 |
| 23 | Detailed Learner tracking is available for discussion posts that were read | Modularity of Availability | 1 | 1 | 1 | 1 |
| 24 | Detailed Learner tracking is available for how long did learners spend on each quiz question | Modularity of Availability | 1 | 1 | 1 | 1 |
| 25 | Detailed Learner tracking available for LMS site activity | Modularity of Availability | 1 | 1 | 1 | 1 |
| 26 | The tracking schedule can be changed by System Admins | Modularity of Availability | 1 | 1 | 1 | 1 |
| 27 | Increased LMS usage (users' load) can be monitored by system admins | Binary Rubric | 1 | 1 | 1 | 1 |
| 28 | Increased LMS usage can be scaled by system admins | Modularity of Availability | 0.66 | 1 | 1 | 1 |
| 29 | Ability to use the command line to create backups in batch. | Modularity of Availability | 1 | 1 | 1 | 1 |
| 30 | Ability to use GUI to create backups in batch. | Modularity of Availability | 1 | 1 | 1 | 1 |
| 31 | The ability for .bak archives to be restored in the LMS with enrolled users | Modularity of Availability | 1 | 1 | 1 | 1 |
| 32 | The ability for .bak archives to be restored in the LMS without enrolled users | Modularity of Availability | 1 | 1 | 1 | 1 |
| 33 | Ability to restore Learner artifacts in a course if a formerly withdrawn Learner re-enrolls. | Modularity of Availability | 0.66 | 1 | 1 | 1 |
| 34 | If a course is deleted mistakenly, it can be restored. | Modularity of Availability | 0.66 | 0.66 | 1 | 1 |
| 35 | Ability to create archives in a proprietary format. | Modularity of Availability | 0.66 | 0.66 | 1 | 1 |
| 36 | Ability to create archives in an open format. | Modularity of Availability | 1 | 0.66 | 1 | 1 |
| 37 | Ability to assign privileges to system roles (various administration-level) | Binary Rubric | 1 | 1 | 1 | 1 |
| 38 | Ability to assign privileges to course roles (various instructor levels) | Binary Rubric | 1 | 1 | 1 | 1 |
| 39 | Ability to assign admins to a selected group of courses (course coordinators) | Modularity of Availability | 1 | 0.66 | 1 | 1 |
| 40 | A configurable catalog that allows you to determine what type of information to display for authenticated users | Modularity of Availability | 1 | 1 | 1 | 1 |
| 41 | LMS is itself a standard-compliant | Binary Rubric | 1 | 1 | 1 | 1 |
| 42 | Interoperability with content that is NOT standards-compliant | Binary Rubric | 1 | 1 | 1 | 1 |

**Table 5- Evaluation for Security**

| # | Features and Functions | Rubric Description | Moodle | Blackboard | TalentLMS | Canvas |
| --- | --- | --- | --- | --- | --- | --- |
| 1 | User IDs and Password determine LMS authentication | Binary Rubric | 1 | 1 | 1 | 1 |
| 2 | Automated system to reset passwords which have forgotten their passwords and/or user IDs | Binary Rubric | 1 | 1 | 1 | 1 |
| 3 | Server-level user authentication | Binary Rubric | 1 | 1 | 1 | 1 |
| 4 | Network-level user authentication | Binary Rubric | 1 | 1 | 1 | 1 |
| 5 | Configurable password security settings | Binary Rubric | 1 | 1 | 1 | 1 |
| 6 | Login encryption/validation | Binary Rubric | 1 | 1 | 1 | 1 |
| 7 | The ability of LMS to allow browser lockdown techniques to ensure Quiz/Exam security. | Binary Rubric | 1 | 1 | 1 | 1 |
| 8 | The ability of LMS to allow proctor/examine supervisor passwords to ensure assessment security | Binary Rubric | 1 | 1 | 1 | 1 |
| 9 | Ability to support SSO from a Learner Portal | Modularity of Availability | 0.66 | 0.66 | 0.66 | 1 |
| 10 | Ability to support SSO from a faculty Portal | Modularity of Availability | 0.66 | 0.66 | 0.66 | 1 |
| 11 | Ability to support external authentication | Modularity of Availability | 0.66 | 1 | 0.33 | 1 |
| 12 | Ability to support internal authentication | Modularity of Availability | 1 | 1 | 1 | 1 |
| 13 | Data and session activity encrypted | Binary Rubric | 1 | 1 | 1 | 1 |
| 14 | the enforcement of strong passwords | Binary Rubric | 1 | 1 | 1 | 1 |
| 15 | the enforcement of password changes | Binary Rubric | 1 | 1 | 1 | 1 |

**Table 6 - Evaluation for Reporting**

| # | Features and Functions | Rubric Description | Moodle | Blackboard | TalentLMS | Canvas |
| --- | --- | --- | --- | --- | --- | --- |
| 1 | LMS tools status can be tracked by LMS administrative reports | Modularity of Availability | 1 | 1 | 1 | 1 |
| 2 | Users' status can be tracked by LMS administrative report | Modularity of Availability | 1 | 1 | 1 | 1 |
| 3 | Customized/User defined reports are supported | Modularity of Availability | 1 | 1 | 1 | 1 |
| 4 | LMS includes roll-up reporting | Modularity of Availability | 1 | 1 | 1 | 1 |
| 5 | LMS includes drill-down reports | Modularity of Availability | 1 | 1 | 1 | 1 |
| 6 | LMS include summary reports | Modularity of Availability | 1 | 1 | 1 | 1 |
| 7 | Reports results can be sorted w.r.t headings/category | Modularity of Availability | 1 | 1 | 1 | 1 |
| 8 | Reports can be scheduled to run automatically | Modularity of Availability | 1 | 1 | 1 | 1 |
| 9 | Reports results can be atomically emailed to specific users | Modularity of Availability | 1 | 1 | 1 | 1 |
| 10 | Data filtering capability of reports is available online | Modularity of Availability | 1 | 1 | 1 | 1 |
| 11 | "out-of-the-box" (OOTB or off the shelf) reports | Modularity of Availability | 1 | 1 | 1 | 1 |
| 12 | Reports to track Instructor-lead courses | Modularity of Availability | 1 | 1 | 1 | 1 |
| 13 | Reports to track Online-lead courses | Modularity of Availability | 1 | 1 | 1 | 1 |
| 14 | Reports to track informal training session | Modularity of Availability | 1 | 1 | 1 | 1 |
| 15 | Users' reports from the Google Chrome browser | Modularity of Availability | 1 | 1 | 1 | 1 |
| 16 | Users' reports from the Mozilla Firefox browser | Modularity of Availability | 1 | 1 | 1 | 1 |
| 17 | The web-based reporting interface is available in GUI | Binary Rubric | 1 | 1 | 1 | 1 |
| 18 | Report formats (HTML, .xls, .csv) available | Binary Rubric | 1 | 1 | 1 | 1 |
| 19 | Report formats (.rtf, .doc, PDF) available | Modularity of Availability | 0.33 | 1 | 1 | 1 |
| 20 | Reports can be sorted by fields (i.e., alphabetically or chronological) | Modularity of Availability | 1 | 1 | 1 | 1 |
| 21 | Dynamic reporting (ad hoc) capabilities for admins | Modularity of Availability | 1 | 1 | 1 | 1 |
| 22 | Online filtering capabilities of Report Data | Binary Rubric | 1 | 1 | 1 | 1 |
| 23 | Select evaluated columns to include in a report | Binary Rubric | 1 | 1 | 1 | 1 |
| 24 | LMS allows program-level-activity reporting | Binary Rubric | 1 | 1 | 1 | 1 |
| 25 | LMS allows individual user-level activity reporting | Binary Rubric | 1 | 1 | 1 | 1 |
| 26 | Real-time reporting | Binary Rubric | 1 | 1 | 1 | 1 |
| 27 | Point-in-time reporting | Binary Rubric | 1 | 1 | 1 | 1 |
| 28 | Option to use filters for reports instead of having to create a new report | Binary Rubric | 1 | 1 | 1 | 1 |

**Appendix C**

**Academic Support Features**

**Table 7 - Evaluation for Talent Development and Certificate Tracking**

| # | Features and Functions | Rubric Description | Moodle | Blackboard | TalentLMS | Canvas |
| --- | --- | --- | --- | --- | --- | --- |
| 1 | Learners' Career tracking | Modularity of Availability | 0.66 | 1 | 1 | 1 |
| 2 | Certification Management tool | Modularity of Availability | 0.66 | 1 | 1 | 1 |
| 3 | Competency Management tool | Modularity of Availability | 0.66 | 1 | 1 | 1 |

**Table 8 - Evaluation for Workflow & Approval Process**

| # | Features and Functions | Rubric Description | Moodle | Blackboard | TalentLMS | | Canvas |
| --- | --- | --- | --- | --- | --- | --- | --- |
| 1 | Manage a waitlist queue (approve/deny). | Modularity of Availability | 0.66 | 0.66 | 0.66 | 0.66 | |
| 2 | Learner automatic self-registration option | Modularity of Availability | 1 | 1 | 1 | 1 | |
| 3 | Learner self-registration option with a key | Modularity of Availability | 1 | 1 | 1 | 1 | |
| 4 | Registration with manager approval | Modularity of Availability | 1 | 1 | 1 | 1 | |
| 5 | Registration confirmation via email | Modularity of Availability | 1 | 1 | 1 | 1 | |

**Table 9 - Evaluation for Instructor-led courses**

| # | Features and Functions | Rubric Description | Moodle | Blackboard | TalentLMS | Canvas |
| --- | --- | --- | --- | --- | --- | --- |
| 1 | Instructor-led learning courses can be assigned by the LMS course administrators | Modularity of Availability | 1 | 1 | 1 | 1 |
| 2 | Instructor-led learning courses can be tracked by the LMS course administrators | Modularity of Availability | 0.66 | 1 | 1 | 1 |
| 3 | Proactive tool for conflict checking by course administrators for room and instructor scheduling | Modularity of Availability | 0.66 | 0.66 | 0.66 | 0.66 |
| 4 | The max Learners limit for a course at the session level can be edited/managed by course administrators | Modularity of Availability | 0.66 | 1 | 1 | 1 |
| 5 | Automatic Learners' waitlist functionality if Learners' limit available | Modularity of Availability | 0.66 | 0.66 | 0.66 | 0.66 |
| 6 | Enroll and cancel registration within the registration deadline by course administrators | Modularity of Availability | 1 | 1 | 1 | 1 |
| 7 | Ability to reserve rooms, equipment, and other resources | Modularity of Availability | 0.66 | 0.66 | 0.66 | 0.66 |

**Table 10 - Evaluation for Evaluation Feature in LMS**

| # | Features and Functions | Rubric Description | Moodle | Blackboard | TalentLMS | Canvas |
| --- | --- | --- | --- | --- | --- | --- |
| 1 | LMS has Assessment Tool for assignments | Binary Rubric | 1 | 1 | 1 | 1 |
| 2 | LMS has Assessment Tool for quizzes/exam | Binary Rubric | 1 | 1 | 1 | 1 |
| 3 | Ability to set passing scores for tests | Binary Rubric | 1 | 1 | 1 | 1 |
| 4 | Tests can be auto-graded by the system | Binary Rubric | 1 | 1 | 1 | 1 |
| 5 | Tests can be manual-graded by the course teacher | Binary Rubric | 1 | 1 | 1 | 1 |
| 6 | The ability of LMS to allow IP restrictions to ensure assessment can only be taken in certain locations (campus lab/classrooms) | Modularity of Availability | 1 | 1 | 1 | 1 |
| 7 | The ability of testing tool to randomize quiz question answers | Binary Rubric | 1 | 1 | 1 | 1 |
| 8 | Ability to export grades from LMS to excel | Binary Rubric | 1 | 1 | 1 | 1 |
| 9 | Ability to use rubrics to grade essay questions within an online assessment | Binary Rubric | 1 | 1 | 1 | 1 |
| 10 | Ability to re-grade assessments | Binary Rubric | 1 | 1 | 1 | 1 |
| 11 | Ability to provide detailed tracking of when Learners select an answer (i.e., timestamp of answer choice) within the Assessment tool | Binary Rubric | 1 | 1 | 1 | 1 |
| 12 | Ability to manage what Learners see during and after the quiz submission screen for the result | Binary Rubric | 1 | 1 | 1 | 1 |
| 13 | Ability to grade objective quizzes automatically and reveals results to Learners | Binary Rubric | 1 | 1 | 1 | 1 |
| 14 | Ability to selectively release assessments based on date/time. | Binary Rubric | 1 | 1 | 1 | 1 |
| 15 | The ability for Learners to submit papers online and allow instructors to grade and give feedback without downloading the assignment. | Modularity of Availability | 1 | 1 | 1 | 1 |
| 16 | Ability to export and import from Excel into the Grade Book. | Modularity of Availability | 1 | 1 | 1 | 1 |
| 17 | The ability of a testing tool to allow the use of math symbols. | Binary Rubric | 1 | 1 | 1 | 1 |
| 18 | The ability of a testing tool to allow the use of images. | Binary Rubric | 1 | 1 | 1 | 1 |
| 19 | Ability to grade discussion postings | Binary Rubric | 1 | 1 | 1 | 1 |
| 20 | Ability to grade Chat | Binary Rubric | 1 | 1 | 1 | 1 |
| 21 | Ability to add audio/video components to quizzes or exams. | Binary Rubric | 1 | 1 | 1 | 1 |
| 22 | Ability to drill down to question level to evaluate test questions. | Binary Rubric | 1 | 1 | 1 | 1 |

**Appendix D**

**Content Development and Management**

**Table 11 - Evaluation Table for Learners’ Feature**

| # | Features and Functions | Rubric Description | Moodle | Blackboard | TalentLMS | Canvas |
| --- | --- | --- | --- | --- | --- | --- |
| 1 | Courses can be automatically assigned to Learners without Learners' intervention | Binary Rubric | 1 | 1 | 1 | 1 |
| 2 | LMS notifications are properly structured and notified to Learners in such an order that they cannot miss seeing them | Modularity of Availability | 0.33 | 0.33 | 1 | 1 |
| 3 | Learners can self-enroll in LMS course | Binary Rubric | 1 | 1 | 1 | 1 |
| 4 | Learners' Portal of LMS is user-friendly; every option is clear to read and access | Binary Rubric | 1 | 1 | 1 | 1 |
| 5 | Learners can modify their details on the public profile. | Binary Rubric | 1 | 1 | 1 | 1 |
| 6 | Learners can view online results on LMS | Binary Rubric | 1 | 1 | 1 | 1 |
| 7 | Learners can view courses availability status | Modularity of Availability | 0.33 | 1 | 1 | 1 |
| 8 | Course rating/feedback viewable to Learners. | Modularity of Availability | 0.33 | 0.33 | 1 | 1 |
| 9 | Learners can complete a survey/questionnaire offered throughout LMS | Binary Rubric | 1 | 1 | 1 | 1 |
| 10 | Learners can view a survey/questionnaire results | Binary Rubric | 1 | 1 | 1 | 1 |
| 11 | Learners can access the class/course roster for the enrolled class | Binary Rubric | 1 | 1 | 1 | 1 |
| 12 | Access LMS wikis of the enrolled course | Binary Rubric | 1 | 1 | 1 | 1 |
| 13 | Access discussion forums of the enrolled course | Binary Rubric | 1 | 1 | 1 | 1 |
| 14 | Access chat rooms of the enrolled course | Binary Rubric | 1 | 1 | 1 | 1 |
| 15 | Access LMS wikis throughout LMS (if enabled in a course) | Modularity of Availability | 1 | 1 | 1 | 1 |
| 16 | Access discussion forums throughout LMS (if enabled in a course) | Modularity of Availability | 1 | 1 | 1 | 1 |
| 17 | Access chat rooms throughout LMS (if enabled in a course) | Modularity of Availability | 1 | 1 | 1 | 1 |
| 18 | Learner can create web-based course notes feature | Modularity of Availability | 1 | 1 | 1 | 1 |
| 19 | Learner can self-enroll/un-enroll from any learning offering | Binary Rubric | 1 | 1 | 1 | 1 |
| 20 | Learner can view the waiting list for the approval process | Modularity of Availability | 0.33 | 0.33 | 0.33 | 0.33 |
| 21 | Learner can bookmark online courses | Modularity of Availability | 0.33 | 1 | 0.33 | 1 |
| 22 | Ability to post multimedia (audio/video) feedback for assignments | Modularity of Availability | 1 | 1 | 1 | 1 |
| 23 | Ability to post text-type feedback for assignments | Modularity of Availability | 1 | 1 | 1 | 1 |
| 24 | Ability to post multimedia (audio/video) feedback for discussions activities | Modularity of Availability | 1 | 1 | 1 | 1 |
| 25 | Ability to post text-type feedback for discussions and other Learner activities | Modularity of Availability | 1 | 1 | 1 | 1 |
| 26 | A learner cannot change enrollment status ( self-register, unregister, etc.) after a certain date | Modularity of Availability | 1 | 1 | 0.33 | 1 |
| 27 | The ability for Learners to access LMS profile after graduation (if the administrator enables a user) | Binary Rubric | 1 | 1 | 1 | 1 |

**Table 12 - Evaluation for Learning Content Management**

| # | Features and Functions | Rubric Description | Moodle | Blackboard | TalentLMS | Canvas |
| --- | --- | --- | --- | --- | --- | --- |
| 1 | Course data can be imported into LMS | Modularity of Availability | 1 | 1 | 1 | 1 |
| 2 | Course data can be exported from LMS | Modularity of Availability | 1 | 1 | 1 | 1 |
| 3 | LMS can be used to offer classroom-based courses | Binary Rubric | 1 | 1 | 1 | 1 |
| 4 | LMS can be used to offer web courses | Binary Rubric | 1 | 1 | 1 | 1 |
| 5 | There is a built-in HTML editor | Binary Rubric | 1 | 1 | 1 | 1 |
| 6 | There is a built-in HTML editor to include PDFs and MS Office files | Binary Rubric | 1 | 1 | 1 | 1 |
| 7 | There is a built-in HTML editor to include multimedia files such as audio/videos/etc. | Binary Rubric | 1 | 1 | 1 | 1 |
| 8 | Course content can be uploaded in SCORM/AICC | Modularity of Availability | 1 | 1 | 1 | 1 |
| 9 | Courses can be as a Quiz (Single activity) | Modularity of Availability | 1 | 0.66 | 0.66 | 1 |
| 10 | Courses can be as an Assignment (Single activity) | Modularity of Availability | 1 | 0.66 | 0.66 | 1 |
| 11 | Courses can be as a Survey (Single activity) | Modularity of Availability | 1 | 0.66 | 0.66 | 1 |
| 12 | Courses can be as a Wiki (Single activity) | Modularity of Availability | 1 | 0.66 | 0.66 | 1 |
| 13 | Offline video presentations can be managed by admins on LMS | Modularity of Availability | 1 | 0.66 | 1 | 1 |
| 14 | Online video presentations can be managed by admins on LMS | Modularity of Availability | 1 | 0.66 | 1 | 1 |
| 15 | The single learning activity can be assigned to the entire LMS domain | Modularity of Availability | 1 | 0.66 | 1 | 1 |
| 16 | Bundle learning activities can be assigned in the entire LMS domain | Modularity of Availability | 1 | 1 | 1 | 1 |
| 17 | Courses can be grouped into customized topic areas in the LMS course | Binary Rubric | 1 | 1 | 1 | 1 |
| 18 | Set due dates for course activity | Binary Rubric | 1 | 1 | 1 | 1 |
| 19 | Set due dates for courses itself | Binary Rubric | 1 | 1 | 1 | 1 |
| 20 | Ability to create cloud storage on LMS | Binary Rubric | 1 | 1 | 1 | 1 |
| 21 | Ability to upload cloud storage content on LMS course whenever time it is needed by a teacher | Binary Rubric | 1 | 1 | 1 | 1 |
| 22 | Ability to edit course type/format after making it available for learners | Binary Rubric | 1 | 1 | 1 | 1 |
| 23 | LMS course can be linked to other online resources | Modularity of Availability | 0.66 | 1 | 1 | 1 |
| 24 | Create/edit an Instructor Lead courses | Binary Rubric | 1 | 1 | 1 | 1 |
| 25 | Create/edit an Instructor Lead single activity on LMS | Binary Rubric | 1 | 1 | 1 | 1 |
| 26 | Create/edit without Instructor-lead learning resources | Binary Rubric | 1 | 1 | 1 | 1 |
| 27 | Built-in Learning Content Management System | Binary Rubric | 1 | 1 | 1 | 1 |
| 28 | Quiz Questions include Fill in the Blank type | Modularity of Availability | 1 | 1 | 1 | 1 |
| 29 | Quiz Questions include Short Answer type | Modularity of Availability | 1 | 1 | 1 | 1 |
| 30 | Quiz Questions include the Matching type | Modularity of Availability | 1 | 1 | 1 | 1 |
| 31 | Quiz Questions include Multiple choice type | Modularity of Availability | 1 | 1 | 1 | 1 |
| 32 | Quiz Questions include Essay type | Modularity of Availability | 1 | 1 | 1 | 1 |
| 33 | Quiz Questions include Math-e-Matics type | Modularity of Availability | 1 | 1 | 1 | 1 |
| 34 | Group management features available for creating group assignments | Modularity of Availability | 1 | 1 | 1 | 1 |
| 35 | Ability to create a group discussion topic and then assign it to all groups at once | Modularity of Availability | 1 | 1 | 1 | 1 |
| 36 | Ability to create a group assignment and then assign it to all groups at once | Modularity of Availability | 1 | 1 | 1 | 1 |
| 37 | Ability to change the file size limit on files that can be submitted to the Assignment Tool | Modularity of Availability | 1 | 1 | 1 | 1 |
| 38 | Ability to share content between courses | Binary Rubric | 1 | 1 | 1 | 1 |
| 39 | Ability to easily package a course to import into other repositories | Binary Rubric | 1 | 1 | 1 | 1 |
| 40 | Ability to enable learner and instructor mashups environment | Modularity of Availability | 0.66 | 1 | 1 | 1 |
| 41 | LMS has a journaling tool for learners to upload their journals on LMS | Modularity of Availability | 0.66 | 1 | 1 | 1 |
| 42 | LMS has a journaling tool for learners to read journals on LMS | Modularity of Availability | 0.66 | 1 | 1 | 1 |
| 43 | LMS provides a one-functional equation editor | Modularity of Availability | 1 | 1 | 1 | 1 |
| 44 | LMS has a drag and drop tool to insert file(s) | Modularity of Availability | 1 | 1 | 1 | 1 |
| 45 | LMS utilizes technologies such as AJAX | Modularity of Availability | 0.33 | 0.33 | 0.66 | 1 |
| 46 | Virtual classrooms can be assigned by LMS | Modularity of Availability | 0.66 | 0.66 | 1 | 1 |
| 47 | Online video training can be offered for instructors/staff on LMS | Modularity of Availability | 0.66 | 0.66 | 0.66 | 1 |
| 48 | Training material is available on LMS at no extra cost | Binary Rubric | 1 | 1 | 1 | 1 |
| 49 | The LMS provides prerecorded online training materials | Modularity of Availability | 1 | 1 | 1 | 1 |
| 50 | (Experience)xAPI compliance of LMS | Binary Rubric | 1 | 1 | 1 | 1 |
| 51 | Active Directory Integration with LMS | Modularity of Availability | 1 | 1 | 1 | 1 |
| 52 | Ability to group courses based on group membership (role, location, user profile attributes) for catalog access | Modularity of Availability | 1 | 0.66 | 1 | 1 |
| 53 | Supports embedding content, e.g., YouTube, Slide share, etc. | Binary Rubric | 1 | 1 | 1 | 1 |
| 54 | Single course upload tool | Binary Rubric | 1 | 1 | 1 | 1 |
| 55 | Bulk course upload tool | Binary Rubric | 1 | 1 | 1 | 1 |
| 56 | Ability to support the use of screen readers (example: JAWS). | Modularity of Availability | 1 | 1 | 1 | 1 |
| 57 | Ability to add multiple resources in one category to any course | Binary Rubric | 1 | 1 | 1 | 1 |
| 58 | Disable a course without removing it from the LMS | Binary Rubric | 1 | 1 | 1 | 1 |
| 59 | Set prerequisites for courses | Modularity of Availability | 0.66 | 1 | 1 | 1 |
| 60 | Assign multiple instructors to an LMS course | Binary Rubric | 1 | 1 | 1 | 1 |
| 61 | Ability to embed videos into assessments | Binary Rubric | 1 | 1 | 1 | 1 |
| 62 | LMS has a live whiteboard feature | Modularity of Availability | 0.66 | 0.66 | 0.66 | 1 |

**Table 13 - Evaluation for Learning Content E-library**

| # | Features and Functions | Rubric Description | Moodle | Blackboard | TalentLMS | Canvas |
| --- | --- | --- | --- | --- | --- | --- |
| 1 | Content Library (Uploaded Learning material can be used as references by learners) | Modularity of Availability | 0.66 | 1 | 1 | 1 |
| 2 | Custom Learning Vocabulary/Glossary | Modularity of Availability | 0.66 | 1 | 1 | 1 |
| 3 | LMS has a Wiki tool | Modularity of Availability | 1 | 1 | 1 | 1 |
| 4 | LMS can manage research papers | Modularity of Availability | 0.66 | 1 | 1 | 1 |

**Table 14 - Evaluation for Catalog & Search Feature**

| # | Features and Functions | Rubric Description | Moodle | Blackboard | TalentLMS | Canvas |
| --- | --- | --- | --- | --- | --- | --- |
| 1 | Search interface is clear to use | Binary Rubric | 1 | 1 | 1 | 1 |
| 2 | Search functionality returns results in an easy-to-use combined results view | Binary Rubric | 1 | 1 | 1 | 1 |
| 3 | Advanced search includes a custom field of title | Modularity of Availability | 0.66 | 0.66 | 0.66 | 0.66 |
| 4 | Content tagging by administrator | Modularity of Availability | 1 | 1 | 1 | 1 |
| 5 | Content tagging by learners | Modularity of Availability | 1 | 1 | 1 | 1 |
| 6 | Ability to use search within a course to find course content. | Binary Rubric | 1 | 1 | 1 | 1 |

**Table 15 - Evaluation for Content Plagiarism**

| # | Features and Functions | Rubric Description | Moodle | Blackboard | TalentLMS | Canvas |
| --- | --- | --- | --- | --- | --- | --- |
| 1 | Descriptive Content Plagiarism | Modularity of Availability | 0.66 | 0.66 | 0.66 | 0.66 |
| 2 | Programming Plagiarism | Modularity of Availability | 0.66 | 0.66 | 0.66 | 0.66 |
| 3 | Equation Content Plagiarism | Modularity of Availability | 0.66 | 0.66 | 0.66 | 0.66 |

**Appendix E**

**Smart Features**

**Table 16 - Evaluation for Mobile Features**

| # | Features and Functions | Rubric Description | Moodle | Blackboard | TalentLMS | Canvas |
| --- | --- | --- | --- | --- | --- | --- |
| 1 | Ability to use mobile devices to access course content | Binary Rubric | 1 | 1 | 1 | 1 |
| 2 | See new courses available | Binary Rubric | 1 | 1 | 1 | 1 |
| 3 | Allows Managers to add new users | Binary Rubric | 1 | 1 | 1 | 1 |
| 4 | Ability to launch/create courses | Binary Rubric | 1 | 1 | 1 | 1 |
| 5 | Ability to support administrators in tracking assessments inputs from learners | Binary Rubric | 1 | 1 | 1 | 1 |
| 6 | LMS private messages management tool | Binary Rubric | 1 | 1 | 1 | 1 |
| 7 | Dashboard reporting feature for administrators | Binary Rubric | 1 | 1 | 1 | 1 |
| 8 | All course tools are available in the mobile client | Binary Rubric | 1 | 1 | 1 | 1 |
| 9 | Available tools in the mobile client have the same functionality as the web version | Binary Rubric | 1 | 1 | 1 | 1 |
| 10 | The LMS is mobile-ready when using the web version through smartphones and tablets. | Binary Rubric | 1 | 1 | 1 | 1 |
| 11 | The ability of LMS to send text message alerts | Modularity of Availability | 0.66 | 0.66 | 0.66 | Fully |
| 12 | LMS has a mobile app | Binary Rubric | 1 | 1 | 1 | 1 |
| 13 | Just-in-time (JiT) learning aids availability | Modularity of Availability | 1 | 1 | 1 | 1 |

**Table 17 - Evaluation for Gamification**

| # | Features and Functions | Rubric Description | Moodle | Blackboard | TalentLMS | Canvas |
| --- | --- | --- | --- | --- | --- | --- |
| 1 | Achievement badges | Modularity of Availability | 0.66 | 0.66 | 1 | 1 |
| 2 | Leaderboards by group | Modularity of Availability | 0.66 | 0.66 | 1 | 1 |
| 3 | Leaderboards by class | Modularity of Availability | 0.66 | 0.66 | 1 | 1 |
| 4 | Courses/Content unlocks after achievements | Modularity of Availability | 0.66 | 0.66 | 1 | 1 |
| 5 | Visual aid animation tool | Modularity of Availability | 0.66 | 0.66 | 1 | 1 |
| 6 | Audio effects | Modularity of Availability | 0.66 | 0.66 | 1 | 1 |
| 7 | Experience points (XPs) | Modularity of Availability | 0.66 | 0.66 | 1 | 1 |
| 8 | Customized Challenge Levels | Modularity of Availability | 0.66 | 0.66 | 1 | 1 |
| 9 | Customized groups | Modularity of Availability | 0.66 | 0.66 | 1 | 1 |
| 10 | Participation rate monitoring | Modularity of Availability | 0.66 | 0.66 | 1 | 1 |
| 11 | Content unlocking on achievements | Modularity of Availability | 0.66 | 0.66 | 1 | 1 |
| 12 | User Profiling as Avatars | Modularity of Availability | 0.66 | 0.66 | 1 | 1 |
| 13 | Virtual goods, i.e., helping audio/video/text content availability | Modularity of Availability | 0.66 | 0.66 | 1 | 1 |
| 14 | Virtual goods, i.e., helping a virtual friend | Modularity of Availability | 0.66 | 0.66 | 1 | 1 |
| 15 | Dashboard manager for learners | Modularity of Availability | 0.66 | 0.66 | 1 | 1 |
| 16 | Gamification integration with Facebook | Modularity of Availability | 0.66 | 0.66 | 1 | 1 |
| 17 | Gamification LMS-Independent mode availability | Modularity of Availability | 0 | 0 | 0 | 0 |

**Table 18 - Evaluation for Virtual Classroom**

| # | Features and Functions | Rubric Description | Moodle | Blackboard | TalentLMS | Canvas |
| --- | --- | --- | --- | --- | --- | --- |
| 1 | Disable a room temporarily | Modularity of Availability | 0.66 | 0.66 | 1 | 1 |
| 2 | Create private classes (e.g., only specific users can enroll) | Modularity of Availability | 0.66 | 0.66 | 1 | 1 |
| 3 | Restrict management of individual classes to specific groups or a set of users | Modularity of Availability | 0.66 | 0.66 | 1 | 1 |
| 4 | Enroll and cancel registrations for learning activities | Modularity of Availability | 0.66 | 0.66 | 0.66 | 1 |
| 5 | Create/edit reason to drop Learners from learning activities | Modularity of Availability | 0.66 | 0.66 | 0.66 | 1 |
| 6 | Registration confirmation via email | Modularity of Availability | 0.66 | 0.66 | 0.66 | 1 |
| 7 | Real-time processing capabilities of audio content in a Virtual classroom | Modularity of Availability | 0.66 | 0.66 | 1 | 1 |
| 8 | Real-time processing capabilities of video content in a Virtual classroom | Modularity of Availability | 0.66 | 0.66 | 1 | 1 |
| 9 | Real-time processing capabilities of file-based content in a Virtual classroom | Modularity of Availability | 0.66 | 0.66 | 1 | 1 |
| 10 | Audio conferencing | Modularity of Availability | 0.66 | 0.66 | 1 | 1 |
| 11 | Video conferencing | Modularity of Availability | 0.66 | 0.66 | 1 | 1 |
| 12 | Whiteboard feature includes document sharing | Modularity of Availability | 0.66 | 0.66 | 0.66 | 1 |
| 13 | Whiteboard feature includes document highlighting/pointing | Modularity of Availability | 0.66 | 0.66 | 0.66 | 1 |
| 14 | Instructors can connect to individual Learners | Modularity of Availability | 0.66 | 0.66 | 1 | 1 |
| 15 | An instructor can connect to a group of Learners | Modularity of Availability | 0.66 | 0.66 | 1 | 1 |

**Appendix F**

**Learners' Support Features**

**Table 19 - Evaluation for Administration of Learner Management**

| # | Features and Functions | Rubric Description | Moodle | Blackboard | TalentLMS | Canvas |
| --- | --- | --- | --- | --- | --- | --- |
| 1 | The course registration process is easy to manage by course administrators/teachers | Modularity of Availability | 0.66 | 0.66 | 1 | 1 |
| 2 | Learners' Attendance module is available | Modularity of Availability | 1 | 1 | 1 | 1 |
| 3 | Users with appropriate security rights can assign courses to individual Learners or groups of Learners | Modularity of Availability | 1 | 1 | 1 | 1 |
| 4 | Ability to limit access to specific data and functions based on security rights associated with the user role in the system | Modularity of Availability | 1 | 1 | 1 | 1 |
| 5 | Learners can manually Enroll and un-enroll in the LMS course | Modularity of Availability | 1 | 1 | 1 | 1 |
| 6 | Bulk Learners can be added in LMS | Modularity of Availability | 1 | 1 | 1 | 1 |
| 7 | Bulk Learners can be added to courses | Modularity of Availability | 1 | 1 | 1 | 1 |
| 8 | Activate/deactivate users from the system | Binary Rubric | 1 | 1 | 1 | 1 |
| 9 | Learners' Individual course grades/achievements monitoring | Modularity of Availability | 1 | 1 | 1 | 1 |
| 10 | Instructor Scheduling for online counseling | Modularity of Availability | 0.66 | 0.66 | 1 | 1 |
| 11 | Instructor Scheduling for offline counseling | Modularity of Availability | 1 | 1 | 1 | 1 |
| 12 | Designate/change a learners' LMS course teacher | Binary Rubric | 1 | 1 | 1 | 1 |
| 13 | Ability to map learning outcomes to assessments so that achievement of specific outcomes can be evaluated. | Modularity of Availability | 0.66 | 0.66 | 1 | 1 |
| 14 | Calendar to easily find and enroll in live events | Modularity of Availability | 1 | 0.66 | 1 | 1 |
| 15 | The ability for course admins to combine sections of the same offered course | Binary Rubric | 1 | 1 | 1 | 1 |
| 16 | The ability for course admins to un-combine sections | Binary Rubric | 1 | 1 | 1 | 1 |

**Table 20 - Evaluation for Social Media Features**

| # | Features and Functions | Rubric Description | Moodle | Blackboard | TalentLMS | Canvas |
| --- | --- | --- | --- | --- | --- | --- |
| 1 | Learners can view other Learners on LMS | Modularity of Availability | 1 | 1 | 1 | 1 |
| 2 | Ability to integrate with Facebook | Modularity of Availability | 0.66 | 1 | 1 | 1 |
| 3 | Ability to integrate with Twitter | Modularity of Availability | 0.66 | 1 | 1 | 1 |
| 4 | Ability to integrate with YouTube | Modularity of Availability | 0.66 | 1 | 1 | 1 |
| 5 | LMS social environment is secure w.r.t administrator-defined privacy settings | Modularity of Availability | 0.66 | 1 | 1 | 1 |
| 6 | LMS social environment is secure w.r.t user-defined privacy settings | Modularity of Availability | 0.66 | 1 | 1 | 1 |
| 7 | RSS Feeds availability in the social environment of LMS | Modularity of Availability | 0.66 | 0.66 | 0.66 | 1 |
| 8 | Ability to use RSS feeds to send 0tifications of updated content to Learners | Binary Rubric | 1 | 1 | 1 | 1 |
| 9 | Online interaction with active LMS users on LMS forums | Modularity of Availability | 1 | 1 | 1 | 1 |
| 10 | Online interaction with messaging | Modularity of Availability | 1 | 1 | 1 | 1 |
| 11 | LMS has a Blog tool | Modularity of Availability | 1 | 1 | 1 | 1 |
| 12 | Learners can publish online articles | Modularity of Availability | 1 | 1 | 1 | 1 |
| 13 | Wikis can be contributed by LMS users | Modularity of Availability | 1 | 1 | 1 | 1 |
| 14 | Event Manager/Calendar is viewable | Modularity of Availability | 1 | 1 | 1 | 1 |
| 15 | Polls/Survey can be created by Instructors | Modularity of Availability | 1 | 1 | 1 | 1 |
| 16 | Polls/Survey can be rated by Learners | Modularity of Availability | 1 | 1 | 1 | 1 |
| 17 | Course ratings can be scored by LMS course Learners | Modularity of Availability | 0.66 | 1 | 1 | 1 |
| 18 | Files can be shared on messages | Binary Rubric | 0 | 0 | 0 | 0 |
| 19 | Files can be shared on chat groups | Binary Rubric | 0 | 0 | 0 | 0 |
| 20 | Files can be shared on discussion forums | Binary Rubric | 1 | 1 | 1 | 1 |
| 21 | LMS has a live chat feature | Modularity of Availability | 1 | 1 | 1 | 1 |
| 22 | Ability to log/save chat transcripts by administrators | Binary Rubric | 1 | 1 | 1 | 1 |
| 23 | Threaded discussions are allowed | Modularity of Availability | 1 | 1 | 1 | 1 |
| 24 | Instructor ratings can be scored by LMS course Learners | Modularity of Availability | 0.66 | 0.66 | 1 | 1 |
| 25 | Visible user profiles | Modularity of Availability | 1 | 1 | 1 | 1 |

**Table 21 - Evaluation for Communication & Collaboration Features**

| # | Features and Functions | Rubric Description | Moodle | Blackboard | TalentLMS | Canvas |
| --- | --- | --- | --- | --- | --- | --- |
| 1 | Notifications via Email | Modularity of Availability | 1 | 1 | 1 | 1 |
| 2 | Notifications via SMS | Modularity of Availability | 0.66 | 1 | 1 | 1 |
| 3 | Automated reminder email for learners | Modularity of Availability | 1 | 1 | 1 | 1 |
| 4 | Moderated Discussion groups | Modularity of Availability | 1 | 1 | 1 | 1 |
| 5 | Un-moderated Discussion groups | Modularity of Availability | 1 | 1 | 1 | 1 |
| 6 | Course-specific news section | Modularity of Availability | 1 | 1 | 1 | 1 |
| 7 | Text chat capability | Modularity of Availability | 1 | 1 | 1 | 1 |
| 8 | LMS messaging within two learners | Modularity of Availability | 1 | 1 | 1 | 1 |
| 9 | The ability for Learners to receive announcements outside the LMS for emergency notifications. | Modularity of Availability | 0.66 | 1 | 1 | 1 |
| 10 | The ability for Learners to view announcements within the LMS. | Binary Rubric | 1 | 1 | 1 | 1 |
| 11 | Ability to set start and stop times for calendar events. | Modularity of Availability | 1 | 1 | 1 | 1 |
| 12 | Calendar events can be added automatically from any course activity that has a due date | Binary Rubric | 1 | 1 | 1 | 1 |
| 13 | The ability for Learners to see who is logged into courses | Binary Rubric | 1 | 1 | 1 | 1 |
| 14 | The ability for teachers to see who is logged into courses | Binary Rubric | 1 | 1 | 1 | 1 |
| 15 | Ability to archive incoming and outgoing messages communications throughout the course. | Binary Rubric | 1 | 1 | 1 | 1 |
| 16 | Ability to use search capabilities in internal messages. | Binary Rubric | 1 | 1 | 1 | 1 |
| 17 | Calendaring and scheduling integration with Outlook | Modularity of Availability | 0.33 | 1 | 1 | 1 |
| 18 | Send emails to the user's email address listed in their profile | Modularity of Availability | 1 | 1 | 1 | 1 |
| 19 | Live webcasting capabilities | Modularity of Availability | 0.66 | 1 | 1 | 1 |
| 20 | Live class capabilities | Modularity of Availability | 0.66 | 1 | 1 | 1 |

**Appendix G**

**LMS Support Tools**

**Table 22 - Evaluation for LMS Plugins**

| # | Features and Functions | Rubric Description | Moodle | Blackboard | TalentLMS | Canvas |
| --- | --- | --- | --- | --- | --- | --- |
| 1 | Themes | Modularity of Availability | 1 | 1 | 1 | 1 |
| 2 | Administration Tools | Modularity of Availability | 1 | 1 | 1 | 1 |
| 3 | Collaboration | Modularity of Availability | 1 | 1 | 1 | 1 |
| 4 | Assessments | Modularity of Availability | 1 | 1 | 1 | 1 |
| 5 | Virtual Classroom | Modularity of Availability | 1 | 1 | 1 | 1 |
| 6 | Content Management | Modularity of Availability | 1 | 1 | 1 | 1 |
| 7 | Security | Modularity of Availability | 1 | 1 | 1 | 1 |

**Table 23 - Evaluation for E-commerce Features**

| # | Features and Functions | Rubric Description | Moodle | Blackboard | TalentLMS | Canvas |
| --- | --- | --- | --- | --- | --- | --- |
| 1 | Integrated e-commerce capability for payment of degree/training courses (ILT, OLT, webcast, etc.). | Modularity of Availability | 0.66 | 1 | 1 | 1 |
| 2 | LMS has a feature to buy a course via e-commerce | Modularity of Availability | 0.66 | 1 | 1 | 1 |
| 3 | Discount features (and can differ across domains) available for LMS | Modularity of Availability | 0.66 | 1 | 1 | 1 |
| 4 | Payment system for tracking internal (user's) transaction | Modularity of Availability | 0.66 | 1 | 1 | 1 |
| 5 | LMS has a feature to track e-commerce administrative transactions | Modularity of Availability | 0.66 | 1 | 1 | 1 |

**Table 24 - Evaluation for Technical Support**

| # | Features and Functions | Rubric Description | Moodle | Blackboard | TalentLMS | Canvas |
| --- | --- | --- | --- | --- | --- | --- |
| 1 | Help Desk options available for administrators | Modularity of Availability | 1 | 1 | 1 | 1 |
| 2 | Help Desk options available for Learners | Modularity of Availability | 1 | 1 | 1 | 1 |
| 3 | Email Support for LMS users | Modularity of Availability | 1 | 1 | 1 | 1 |
| 4 | Online chat interaction | Modularity of Availability | 1 | 1 | 1 | 1 |
